# Artificial intelligence-enabled prenatal ultrasound for the detection of fetal cardiac abnormalities: a systematic review and meta-analysis

**DOI:** 10.1016/j.eclinm.2025.103250

**Published:** 2025-05-30

**Authors:** Elena D'Alberti, Olga Patey, Carolyn Smith, Bojana Šalović, Netzahualcoyotl Hernandez-Cruz, J. Alison Noble, Aris T. Papageorghiou

**Affiliations:** a Nuffield Department of Women's & Reproductive Health, University of Oxford, Oxford, United Kingdom; b Department of Maternal and Child Health and Urological Sciences, Sapienza University of Rome, Rome, Italy; c Royal Brompton and Harefield Hospitals, Guy's and St Thomas' NHS Foundation Trust, London, United Kingdom; d Bodleian Health Care Libraries, University of Oxford, Oxford, United Kingdom; e St George's University of London, London, United Kingdom; f Institute of Biomedical Engineering, Department of Engineering Science, University of Oxford, Oxford, United Kingdom

**Keywords:** Congenital heart defect, Fetal ultrasound, Echocardiography, Artificial intelligence, Diagnostic accuracy

## Abstract

**Background:**

Advances in artificial intelligence (AI) have triggered interest in using intelligent systems to improve prenatal detection of fetal congenital heart defects (CHDs). Our aim is to systematically examine the current literature on diagnostic performance of AI-enabled prenatal cardiac ultrasound.

**Methods:**

This systematic review and meta-analysis was registered with PROSPERO (CRD42024549601). Embase, Medline, Cochrane Central Database of Controlled Trials, and CINAHL were searched from inception until February 2025. Studies evaluating AI performance in prenatal detection of fetal CHDs were eligible for inclusion, and studies focusing on the application of AI before 16 weeks of gestation, or using three- or four-dimensional ultrasound, were excluded. Pooled sensitivity and specificity were obtained using random-effect method, and pooled proportions using the Freeman-Tukey arcsine square root transformation. Heterogeneity was assessed with I^2^ statistics. Risk of bias and adherence to reporting standards were assessed using QUADAS-2 and TRIPOD+AI, respectively. Risk of publication bias was assessed with Deek's test and certainty of evidence for outcomes with GRADE approach.

**Findings:**

Fifteen studies were included, of which fourteen developed and evaluated a model and one externally evaluated a previously trained model. Images and videos obtained during cardiac screening or fetal echocardiography of 30.121 fetuses were used for training, validation and testing. For the binary task of classifying heart as normal or abnormal, AI models achieved a pooled sensitivity of 0.89 (95% CI 0.83–0.93, I^2^ = 77.92%) and specificity of 0.91 (95% CI 0.84–0.95, I^2^ = 77.92%). The subgroup analysis showed that models tested on various CHDs exhibited lower sensitivity compared to those tested for a specific cardiac abnormality (0.85; 95% CI 0.75–0.91 vs 0.92; 95% CI 0.87–0.96), while specificity remained comparable (0.90; 95% CI 0.79–0.96 vs 0.91; 95% CI 0.81–0.97). Overall, AI models performed better than operators with lower expertise and were nearly comparable to experts; however, the human comparator group (median six clinicians, IQR 3–10) was usually small and non-blinded. Relevant sources of heterogeneity were the types of cardiac views collected, the prevalence of CHDs across different datasets, and the types of CHDs examined. The risk of bias was moderate-high and adherence to reporting standards low (>70% in 18/51 TRIPOD+AI items). The risk of publication bias was not statistically significant (Deek's test p = 0.474).

**Interpretation:**

These findings suggest that AI models perform better than clinicians with lower expertise, but this must be interpreted with caution due to the high risk of bias and sources of heterogeneity.

**Funding:**

This study was partly supported by the InnoHK-funded Hong Kong Centre for Cerebro-cardiovascular Health Engineering (COCHE) Project 2.1 (Cardiovascular risks in early life and fetal echocardiography). ATP and JAN are supported by the 10.13039/501100000272National Institute for Health and Care Research (NIHR) 10.13039/501100013373Oxford Biomedical Research Centre (BRC).

**Research in context**

**Evidence before this study**

The existing evidence on prenatal detection of congenital heart defects (CHDs) using ultrasound shows low detection rates in clinical practice; studies in the field using Artificial Intelligence (AI) to improve detection were noted. No meta-analyses had been performed to assess pooled estimates of AI diagnostic performance for prenatal CHD detection prior to this study. A focused analysis was therefore undertaken to examine the value of AI models for the detection of CHDs. Databases including Embase, Medline, Cochrane Central Database of Controlled Trials, and CINAHL were searched from inception to February 14, 2025. Studies involving AI models applied to 2D ultrasound cardiac screening or fetal echocardiography between 16 and 40 weeks gestation were included. The existing evidence suggests that AI model performance is promising in identifying normal cardiac structures and performing segmentation, but limited data were available on classifying normal vs abnormal fetal hearts or detecting specific CHDs.

**Added value of this study**

This study systematically reviewed and analysed the diagnostic accuracy of AI-enabled prenatal cardiac ultrasound in detecting CHDs, providing pooled sensitivity and specificity estimates. It is the first review to compare AI model diagnostic performance against clinicians in real-world clinical settings and to evaluate AI model readiness for clinical implementation. The findings demonstrate important sources of heterogeneity in datasets, differences of cardiac views and types of CHD across studies. We highlight limitations and strengths of current AI models, including the potential for AI models to outperform less experienced operators–while achieving near-expert accuracy in some cases.

**Implications of all the available evidence**

While AI-enabled models hold potential to improve prenatal CHD detection, their clinical implementation faces several challenges. Although they could enhance the accuracy of detection, data are currently lacking in integration of AI models into routine practice in assistive-AI tools. Prospective studies, particularly in the most relevant community screening settings and resource-limited settings, are needed.

## Introduction

Globally, the leading causes of infant mortality are—in order—prematurity-related illnesses, adverse intrapartum events, and congenital abnormalities.^1^ Of the latter, congenital heart defects (CHDs) are the most common structural abnormalities at birth, affecting nearly 0.8% of live births.^2^^,^^3^ In addition to being common, CHDs are associated with high mortality and morbidity rates; this combination of high prevalence and high rates of adverse outcome makes them the leading cause of infant mortality due to congenital abnormalities.^2^, ^3^, ^4^

Infants diagnosed prenatally with severe CHDs, as those with ductal-dependent circulation, have better postnatal outcomes and survival rates than those identified after birth.^5^ Mortality rates prior to cardiac surgery and post-operative survival are more favourable in infants with critical defects, such as coarctation of the aorta or transposition of the great arteries, detected in *utero* rather than postnatally (preoperative mortality 0.3% vs 3.0% and postoperative survival 99.3% vs 97.0%, respectively).^5^ Thus, given the importance of prenatal detection, pregnant individuals in most high-income countries (HICs) are offered screening for CHDs as part of the routine mid-trimester anatomical ultrasound scan.^6^, ^7^, ^8^ This allows optimal perinatal management such as birth in a unit with expert cardiac support available.^9^ Hence, planned management to achieve neonatal hemodynamic stability allows the prevention of hypoxia, acidosis and related neonatal morbidity, reducing ventilation time and the risk of neurological injury.^10^

Despite the evident importance of prenatal detection, data from HICs indicates that the policy of universal cardiac screening still fails to recognise nearly half of those affected with a CHD.^11^ Efforts have relied on the implementation of protocolised screening,^12^ and benefitted from improvements in ultrasonographic imaging clarity^13^; nevertheless, the screening performance of routine cardiac assessment remains poor, particularly in underserved settings, where resource constraints may result in a lack of experienced operators. Referral for formal fetal echocardiography—a comprehensive ultrasonographic evaluation of the heart by subspecialist fetal cardiologists—has a high diagnostic performance,^14^ but is not possible for all pregnant individuals: only about 10% of individuals at high-risk of fetal CHDs are referred for this examination after a screening exam, and this rate is based on the availability of such specialists and cost-effectiveness.^15^^,^^16^

The importance of CHD detection, limitations of current screening, and the emergence of artificial intelligence (AI)-based models applied to clinical ultrasound have led naturally to the question: could AI-models be useful as tools to improve prenatal diagnosis of fetal CHDs?^17^ Initial data have been promising, with AI successfully achieving automated recognition of normal cardiac views and segmentation of specific heart structures, with good agreement with human operators.^18^ Fewer studies have examined the more clinically useful task—investigating the correct prenatal classification of the normal vs abnormal heart, or the recognition of specific CHDs.

The aim of this study is to systematically review the current knowledge of the diagnostic performance of AI-enabled prenatal cardiac ultrasound, and to compare this to clinical human performance, in order to evaluate readiness of published work for potential clinical implementation, based on the risk of bias and adherence to TRIPOD+AI reporting standards.^19^

## Methods

### Search strategy

This systematic review and meta-analysis was conducted using guidance from the Cochrane Handbook for Systematic Reviews of Interventions, and following an a-priori designed protocol proposed by the Meta-analyses of Observational Studies in Epidemiology (MOOSE) group.^20^^,^^21^ Findings were reported according to the Preferred Reporting Items for Systematic Reviews and Meta-analyses (PRISMA) guidelines,^22^ and the PRISMA checklist was completed and provided as Supplementary Materials.^22^ Prior to commencing this review, a study protocol was developed and registered with the PROSPERO International Prospective Register of Systematic Reviews (CRD42024549601).

Embase and Medline were electronically searched on OVID [1974-present and 1946-present, respectively], while Cochrane Central Database of Controlled Trials [Issue 7 of 12, July 2024] and CINAHL [1981-present] on their websites, from inception to July 4, 2024, using free-text keywords and subject headings based on the search reported in the Appendix. All the databases were searched separately, with similar but adapted search strategies. A search update was run up to February 14, 2025, during the manuscript revision process to ensure the inclusion of the most recent evidence. No limits were placed on the searches as carried out in the databases as any limits were applied after, using the inclusion and exclusion criteria as set out in the protocol. No published search filters were used. All search strategies were devised by the information specialist (CS) for this systematic review only and have not been used previously elsewhere. The search strategy was reviewed and approved by the lead authors before being run on the databases. In order to deduplicate, all records were uploaded to EndNote 21 and each set of duplicate results was carefully examined to ensure that false hits were not removed.

### Study selection and eligibility criteria

Two independent reviewers (E.D. and O.P.) selected the studies in stages by first reviewing titles and abstracts of results obtained from the search to identify potentially relevant studies. Full-text articles were subsequently evaluated to determine their eligibility for inclusion. The reference lists of all eligible studies were screened manually for additional citations not identified by the initial electronic search. Agreement regarding inclusion and exclusion of studies was achieved by consensus between the two reviewers or by consultation with a third reviewer (A.T.P.). Literature reviews, conference abstracts, case reports with less than five subjects, editorials, letters and personal communications were excluded.

**Fig. 1:**
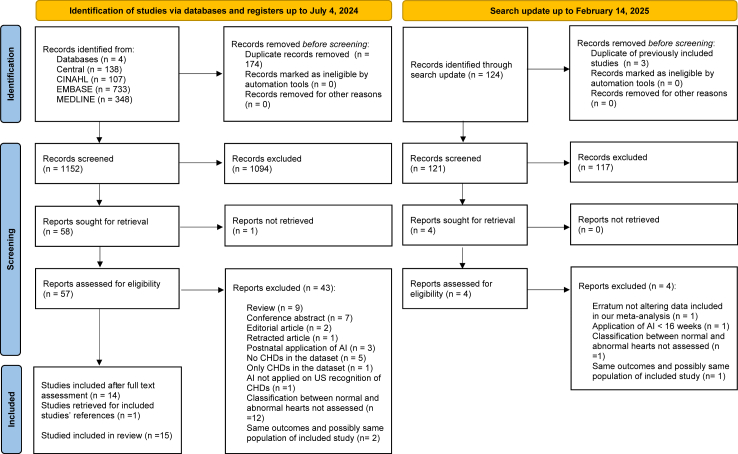
**PRISMA flowchart**; adapted from PRISMA 2020 checklist^22^; AI, artificial intelligence; CHDs, congenital heart defects; US, ultrasound.

As our systematic review sought to understand applications for most healthcare settings, where routine cardiac assessment is performed around the mid-trimester anatomy scan using two-dimensional (2D) ultrasound, we made an a-priori decision to include those studies reporting on application of AI in 2D ultrasound cardiac screening or fetal echocardiography from 16 to 40 weeks of gestation. Unrestricted criteria were applied to different AI machine-learning approaches, and we considered still images of B-mode or colour-Doppler, and videos or short “sweeps” as data inputs. Similarly, no restriction was placed on the imaging planes (transverse or sagittal). Prospective and retrospective observational studies, evaluating pregnancies with any level of prior risk, were eligible for inclusion, including singleton or multiple gestations, and in any healthcare setting. Studies exclusively focusing on the application of AI before 16 weeks, or on 3D/4D ultrasound, like spatiotemporal image correlation (STIC) techniques, not commonly used in routine screening, were excluded. Moreover, studies focusing on the AI assessment of normal cardiac structures or on segmentation only, without reporting the diagnostic performance for CHDs, were excluded (Table 1). No language restrictions were applied to the search strategy, allowing inclusion of studies in any language. Every attempt was made to identify publications from the same research groups that shared screened subjects for the same CHDs. In such cases, only the study judged to be the most relevant to the aims of the present review, or the one with the largest cohort was included (Table 1). The flowchart of the literature search is presented in Fig. 1.

**Table 1:** Inclusion and exclusion criteria according to PICOS criteria.

PICOS criteria	Inclusion criteria	Exclusion criteria
Population	Singletons or twins with both normal and abnormal fetal heart evaluated from 16 weeks' gestation onwards in community screening departments or fetal cardiology units	Singletons or twins with both normal and abnormal heart examined before 16 weeks' gestation
Intervention	AI-enabled 2D cardiac ultrasound with unrestricted criteria applied to the: - Type of examinations: fetal echocardiography or cardiac screening; - Type of US settings: still images of B-mode or colour-Doppler, and cine-loops or sweeps; - Type of imaging planes: axial or sagittal.	AI-enabled 3D/4D cardiac ultrasound, like STIC techniques
Comparison	Clinicians' performance (when applicable)	N/A
Outcomes	1) AI classification of normal vs abnormal fetal heart 2) AI detection of specific CHDs	1) AI assessment of normal cardiac structures only 2) AI segmentation, without reporting the diagnostic performance for CHDs
Study design	Both prospective and retrospective studies, evaluating pregnancies with any level of prior risk.	Reviews, conference abstracts, case reports with less than five subjects included, editorials, letters and personal communications.

AI, artificial intelligence; CHDs, congenital heart defects; STIC, spatiotemporal image correlation; N/A, not applicable; US, ultrasound; 2D, two-dimensional; 3D/4D, Three/four-dimensional.

### Data analysis

For each study two independent reviewers (E.D. and B.S.) extracted basic data authors’ name; year of publication; country where the study was conducted, categorised as a HIC or low-middle-income country (LMIC)^23^; study design; the AI model used; the clinical task evaluated; the type of annotation; the presence of a training, validation and testing stage; the type of cardiac assessment (cardiac screening or specialist fetal echocardiography, or both); the gestational age (GA) at the time of cardiac ultrasound; and the healthcare setting where studies were conducted. Details regarding the AI model development and testing were also extracted, including the number of data used for training, validation and testing, split into normal and abnormal cases. The heart views that were used were also recorded, including evaluation of the cardiac situs, the four-chamber views (4CV), the left and right ventricular outflow tracts (LVOT and RVOT, respectively), the three-vessel (3VV) and 3-vessel-trachea (3VT) views, and sagittal views; the use of images, videos, cardiac biometrics and Doppler, including both colour-Doppler and pulsed-wave Doppler. Lastly, we extracted data on diagnostic accuracy metrics provided for the AI models and the clinicians, and the prevalence of CHDs in the datasets used.

### Statistics

The index test was the AI model, and the reference standard was the prenatal diagnosis by experts in fetal cardiology, the postnatal or post-mortem confirmation. Since only one study provided true positive (TP), false positive (FP), false negative (FN) and true negative (TN) cases, a diagnostic test accuracy meta-analysis was performed creating 2 × 2 tables with TP, FP, FN, TN calculated using the number of cases included, the prevalence of CHDs in the study population, and the sensitivity and specificity reported in each study. In studies reporting performance of multiple models, the one with the highest specificity was selected by consensus, as this best aligns with priorities of national prenatal screening programmes to minimize false positives while maintaining diagnostic accuracy. The primary outcome was the performance of AI model classification of normal vs abnormal hearts. A sub-group analysis was performed to assess the performance of AI models when tested for various CHDs or a single cardiac defect. Intended subgroup and meta-regression analysis for the performance of AI by gestational age, in screening settings vs fetal cardiology units, and for specific heart abnormalities was not possible due to limited data. Pooled sensitivity and pooled specificity with 95% Confidence interval (95% CI) were provided using random-effect method and applying a 0.5 continuity correction. We performed a leave-one-out sensitivity analysis to assess the robustness of the results and the potential influence of any single study on the overall effect size. Specifically, the meta-analysis was repeated excluding one study at a time, and the pooled sensitivity and specificity was recalculated for each iteration. In addition, we conducted a secondary sensitivity analysis in which we removed multiple studies identified as having low adherence to reporting guidelines or deemed to be at high risk of bias. This allowed us to examine whether these studies had a disproportionate impact on the overall results. Cochrane RevMan5 was used to create summary ROC curves (sROCs), analysing the AI classification task of normal vs abnormal hearts. Specific sROCs for individual cardiac defects or grouped by shared clinical or ultrasonographic findings were also assessed. For instance, aortic coarctation was assessed combined with duct-dependent CHDs and with hypoplastic left heart syndrome. Estimation of data partitioning with pooled proportions was obtained using the Freeman-Tukey arcsine square root transformation under a random-effect model. The Freeman-Tukey transformation was used to stabilize variance and normalize the distribution of proportions. This method is widely applied in proportion meta-analysis as it ensures a balanced contribution of studies with extreme proportions and provides a robust synthesis under a random-effect model.^24^ Heterogeneity was assessed with I^2^ statistics. To assess the risk of publication bias, we performed Deek's test, that is recommended in diagnostic accuracy meta-analyses to evaluate the presence of small-study effects. A p-value <0.05 suggests significant asymmetry, indicating potential publication bias. All analyses were undertaken using “metadta”, “metandi” and “metan” packages in STATA 18, College Station, TX: StataCorp LLC.

### Assessment of risk of bias, adherence to reporting standards and certainty of evidence

For each study two independent reviewers (E.D. and B.S.) independently assessed the adherence to reporting standards and risk of bias. Assessment of the risk of bias was undertaken for all the included studies based on the Quality Assessment of Diagnostic Accuracy Studies (QUADAS-2) tool using Cochrane RevMan5. This tool evaluates studies within four key domains: patient selection, index test, reference standard and flow of patients through the study. The last item was modified intentionally as the flow of data rather than patients. Each study in the review was graded as having either a low, high or unclear risk of bias for each domain and for lack of applicability based on a series of signalling questions.^25^

We assessed the reporting quality of the included studies guided by Transparent Reporting of a multivariable prediction model for Individual Prognosis Or Diagnosis + Artificial Intelligence (TRIPOD+AI).^19^ The TRIPOD+AI checklist, an updated version of the original TRIPOD, was published in April 2024. Although we recognize that the included studies were either published or submitted for publication before the introduction of TRIPOD+AI, we have adopted this version to align with its objective of harmonising and standardizing the reporting systems for studies employing AI models. TRIPOD+AI comprises 27 items and, including subitems, a total of 52 points. As our systematic review is based on studies assessing the prediction or diagnosis of CHDs that might require surgical interventions postnatally, we evaluated the studies according to 51 total items, excluding one that refers to treatments received during model development (subitem 6c). Furthermore, we assessed studies conducted in a single centre with 49 items, excluding two subitems related to clustering data across multiple hospitals (12 d and 23 b). Lastly, one study externally testing a previously developed AI model was assessed for 45 evaluation items. Analogous to a prior study that employed the original TRIPOD version, studies were assessed based on the percentage of points relative to the total achievable score.^26^ Studies were classified as having low adherence if the score was below 50%.^26^ Moreover, we further defined a moderate adherence, when scores ranged from 50% to 70%, and high adherence for scores exceeding 70%, acknowledging that TRIPOD+AI encompasses a greater number of items and subitems compared to TRIPOD (+40%, 52 vs 37).

The certainty of evidence was assessed using the GRADE (Grading of Recommendations, Assessment, Development, and Evaluations) approach for AI diagnostic accuracy analysis. The evaluation was conducted according to the GRADE Handbook with the assessment of five key domains: risk of bias, using the QUADAS-2 tool; inconsistency, measured by statistical heterogeneity (I^2^) and the stability of pooled estimates in sensitivity analyses; indirectness, based on differences between studies' and real-world population, AI performance real-time vs in retrospect, and quality of dataset compared to real clinical settings; imprecision, according to the CI width for sensitivity and specificity estimate; publication bias, evaluated using Deek's asymmetry test, when applicable. The starting rating for evidence quality was downgraded in any of the five domains in accordance with the Handbook. Two independent raters (E.D. and B.S.) assessed the quality of evidence, with discrepancies resolved through discussion.

### Ethics

This study was conducted using exclusively publicly accessible data and did not involve direct enrolment of human participants, therefore it did not require approval from institutional ethical committee. The methodology adhered to recognized reporting standards for systematic review and meta-analysis, including prior protocol registration and a clearly defined search and analysis strategy to promote transparency.

### Role of the funding source

The funder of the study had no role in study design, data collection, analysis or interpretation, nor in the writing of the paper.

## Results

The electronic search inclusive of the literature update yielded 1.273 citations. Following review of titles and abstracts, sixty-two publications underwent full-text review, of which fifteen studies evaluating AI-enabled ultrasound for prenatal detection of CHDs in 30.121 fetuses were included; all were performed between 2020 and 2024 (Fig. 1).^27^, ^28^, ^29^, ^30^, ^31^, ^32^, ^33^, ^34^, ^35^, ^36^, ^37^, ^38^, ^39^, ^40^, ^41^ Table 2 summarises the characteristics of the included studies. Among the studies there were no randomised controlled trials nor prospective studies of diagnostic effectiveness. The methodology of data collection was clearly reported by twelve studies: ten used only retrospective data,^27^^,^^28^^,^^32^, ^33^, ^34^^,^^36^, ^37^, ^38^^,^^40^^,^^41^ one used prospective^30^ and another one both prospective and retrospective cases.^35^ In the remaining three studies it was unclear whether data collection was performed retrospectively or prospectively,^29^^,^^31^^,^^39^ with two of them mentioning an opt-out design, but no further details.^29^^,^^30^ Six studies used multicentre datasets within the same country^28^^,^^29^^,^^31^^,^^35^^,^^39^^,^^40^ and no multicentre international studies were identified. Data were recorded from cardiac screening,^34^^,^^40^ fetal echocardiography,^27^^,^^32^^,^^33^^,^^36^, ^37^, ^38^^,^^41^ or both,^28^^,^^35^ and four studies did not report on the type of cardiac assessment.^29^, ^30^, ^31^^,^^39^ Eight studies used data from cardiac scans performed between the second and third trimester.^27^^,^^29^, ^30^, ^31^, ^32^^,^^37^^,^^38^^,^^41^ Most studies were conducted in HICs,^27^, ^28^, ^29^^,^^31^^,^^32^^,^^34^, ^35^, ^36^, ^37^, ^38^, ^39^, ^40^, ^41^ and only two in LMICs.^30^^,^^33^ Also, these were performed in tertiary units or university hospitals,^27^, ^28^, ^29^, ^30^, ^31^, ^32^, ^33^^,^^35^, ^36^, ^37^, ^38^, ^39^^,^^41^ except for two in community-screening settings.^34^^,^^40^

**Table 2:** Characteristics of the included studies.

Lead author (year)	Country	Design	AI architecture backbone	Task	Annotation	Training	Validation	Testing	Heart exam	GA	Healthcare setting
Gong 2020^27^	HIC (China)	Retrospective	DGACNN	Classification of normal vs abnormal hearts	Training dataset was partially labelled by expert cardiologists	✓	✓	✓	FE	18–39 weeks	University hospitals
Arnaout 2021^28^	HIC (USA)	Multicenter Retrospective	U-Net; ResNet;	Classification of normal vs abnormal hearts	Training datasets were labeled and manually traced by experts	✓	✓	✓	FE CS	18–24 weeks	Tertiary level units
Komatsu 2021^29^	HIC (Japan)	Multicenter Opt-out design	YOLOv2	Classification of normal vs abnormal hearts	Experts labeled and indicated structures on the training dataset	✓	✓	✓	NR	18–34 weeks	University hospitals
Nurmaini 2022^30^	LMIC (Indonesia)	Prospective	DenseNet201	Classification of normal vs abnormal hearts	Videos annotated by fetal cardiologists	✓	✓	✓	NR	II-III trimester	University hospital
Sakai 2022^31^	HIC (Japan)	Multicenter Opt-out design	YOLOv2	Classification of normal vs abnormal hearts	NR	✓	✓	✓	NR	18–34 weeks	University hospitals
Wang 2022^32^	HIC (China)	Retrospective	DeepLabv3+; PSPNet; FastFCN; DenseASPP;	Prenatal detection of TAPVC	Trained fetal cardiology residents labeled the frames	✓	NR	✓	FE	25.6 ± 2.7 weeks	University hospital
Truong 2022^33^	LMIC (Vietnam)	Retrospective	Random forest	Classification of normal vs abnormal hearts	NR	✓	✓	✓	FE	22.0 (21–24) weeks	Tertiary level unit
Athalye 2023^34^	HIC (The Netherlands)	Retrospective	U-Net; ResNet.	Classification of normal vs abnormal hearts	as Arnaout et al.	X	X	✓	CS	18–22 weeks	Community-screening unit
Tang 2023^35^	HIC (China)	Multicenter Retrospective and Prospective	DDCHD-DenseNet	Classification and prenatal detection of duct-dependent CHDs	NR	✓	✓	✓	FE CS	NR	University hospitals
Day 2023^36^	HIC (UK)	Retrospective	ResNet50	Prenatal detection of HLHS	Images were retrieved by one expert	✓	✓	✓	FE	20^+0^-23^+6^ weeks	Tertiary level unit
Yang 2023^37^	HIC (China)	Retrospective	YOLOv5 ResNet50 MobileNetv2	Classification of normal vs abnormal hearts Prenatal recognition of VSD	NR	✓	✓	✓	FE	19–39 weeks	University hospital
Day 2024^38^	HIC (UK)	Retrospective	ResNet50	Prenatal detection of AVSD	Images were labeled by experts	✓	✓	✓	FE	18^+0^ -32^+0^ weeks	Tertiary level unit
Yang 2024^39^	HIC (China)	Multicenter NR	HOCAD FasterRCNN	Prenatal detection of ST, PAC, AVB	Experienced sonographers (6 years of experienced) manually labelled A and V waves	✓	✓	✓	NR	NR	University hospitals but it is unclear whether scans were performed also at community-screening units
Taksoee-Vester 2024^40^	HIC (Denmark)	Multicenter Retrospective	U-Net	Prenatal detection of CoA	Automatic segmentation manually corrected by one annotator	✓	✓	✓	CS	18–22 weeks	Community-screening unit
Zhou 2024^41^	HIC (China)	Retrospective	U-Net MA-Net Link-Net	Prenatal detection of AVSD	Images were labeled by a junior physician (3–5 years of experience in FE) and reviewed by a physician with 10 years of experience	✓	*NR*	✓	FE	24.4 ± 4.1 weeks	University hospital

AI, artificial intelligence; AVB, atrioventricular block; AVSD, atrio-ventricular septal defect; CHD, congenital heart defects; CoA, coarctation of the aorta; CS, cardiac screening; DenseASPP, Dense Atrous Spatial Pyramid Pooling; DGACNN, model based on a discriminator generative adversarial network (GAN) and CNN layers; FastFCN, Fast Fully Convolutional Network; FE, fetal echo cardiography; GA, gestational age; HIC, high income countries; HLHS, hypoplastic left heart syndrome; HOCAD, hierarchical online contrastive anomaly detection; NR, not reported; PAC, premature atrial contractions; PSPNet, Pyramid Scene Parsing Network; ST, sinus tachycardia; TAPVC, total abnormal pulmonary vein connection; VSD, ventricular septal defect; YOLO, you-only-look-once.

Five studies focused on the 4CV alone,^27^^,^^30^^,^^32^^,^^36^^,^^38^ seven studies used all standard axial views recommended by ISUOG,^7^^,^^28^^,^^29^^,^^31^^,^^33^^,^^37^^,^^40^^,^^41^ and five considered sagittal views.^33^^,^^35^^,^^40^^,^^41^ Moreover, nine studies considered the detection of any CHDs, while six focused on the recognition of specific cardiac abnormalities (Supplementary Table S1), such as total anomalous pulmonary venous connection, duct-dependent CHDs, hypoplastic left heart syndrome, atrioventricular and ventricular septal defects and coarctation of the aorta.^32^^,^^35^, ^36^, ^37^, ^38^^,^^40^^,^^41^ Only one study assessed AI classification of fetal arrhythmia.^39^

In fourteen studies datasets were used to train and test the AI models,^27^, ^28^, ^29^, ^30^, ^31^, ^32^, ^33^^,^^35^, ^36^, ^37^, ^38^, ^39^, ^40^, ^41^ while one study externally tested a previously trained algorithm.^34^ The performance of AI models compared to clinicians was reported by eleven studies,^27^^,^^28^^,^^30^, ^31^, ^32^^,^^34^, ^35^, ^36^^,^^38^^,^^39^^,^^41^ (Supplementary Table S2). Studies excluded from our analysis and reasons for exclusion are listed in Supplementary Table S3.

### Model development and testing

Details related to development and testing of AI models and data partitioning for training, validation and testing are summarised in Table 3. Validation methods were reported by six studies, with three of them using split sample, with the available dataset divided into two sets, one to develop the model and the other to validate it, and cross-validation, respectively. One study conducted an external evaluation of a previously trained model in a different country, using data collected from a screening unit, with 40% of CHDs that were not included in the dataset used for the initial model's development.^34^

**Table 3:** Protocol for AI model development and/or testing.

Study (year)	N. Fetuses	Training (normal)	Training (abnormal)	Validation (normal)	Validation (abnormal)	Testing (normal)	Testing (abnormal)	Heart views	Images	Videos	Cardiac biometrics	Doppler	TRIPOD+AI adherence
Gong 2020^27^	NR	2.655 images	541 images	200 images	200 images	200 images^a^	200 images^a^	**Axial view:** 4CV	X	✓	X	X	**LOW (29.4%)**
Arnaout 2021^28^	5.867	69.841 images	102.974 images	NR	NR	**FETAL-125** 11.445 images **OB-125** 220.990 images **OB-4000** 4.365.437 images **BCH-400** 4.389 images **TWINS-10** NR the amount of normal images	**FETAL-125** 8.377 images **OB-125** 108.415 images **OB-4000** 108.415 images **BCH-400** 40.123 images **TWINS-10** NR the amount of abnormal images	**Axial views**: Situs, 4CV, LVOT, RVOT, 3VV, 3VT (axial sweeps from BCH-400) **Sagittal views:** LVOT	✓	✓	✓	X	**HIGH (70.6%)**
Komatsu 2021^29^	363	668 videos	X	10 videos	10 videos	42 videos	42 videos	**Axial sweeps** from situs to 3VT	X	✓	X	X	**MODERATE (54.9%)**
Nurmaini 2022^30^	76	157 images	812 images	NR	NR	**Testing intra-patient** 20 images **Testing inter-patient** 5 images	**Testing intra-patient** 140 images **Testing inter-patient** 50 images	**Axial views** 4CV	✓	X	X	X	**LOW (44.9%)**
Sakai 2022^31^	160	292 videos	X	6 videos	6 videos	20 videos	20 videos	**Axial sweeps** from situs to 3VT	X	✓	X	X	**MODERATE (56.9%)**
Wang 2022^32^	319	492 images	48 images	X	X	82 images	20 images	**Axial view:** 4CV	X	✓	✓	✓ PWD, CD	**MODERATE (51.0%)**
Truong 2022^33^	3.910	NR	NR	NR	NR	NR	NR	**Axial and sagittal views:** Comprehensive FE	NR	NR	✓	✓ PWD	**LOW (42.8%)**
Athalye 2023^34^	108	Previously trained (Arnaout 2021)	Previously trained (Arnaout 2021)	42 cases (NR the amount of images)	66 cases (NR the amount of images)	Previously tested (Arnaout 2021)	Previously tested (Arnaout 2021)	**Axial views:** 4CV, LVOT, 3VV and 3VT	✓	X	X	X	**MODERATE (60%)**
Tang 2023^35^	6.941	4.018 images	2.694 images	191 images	163 images	200 images	150 images	**Sagittal view:** Ao arch	✓	✓	X	X	**MODERATE (52.9%)**
Day 2023^36^	161	5.019 images	3.241 images	*593 images*	*380 images*	676 images	339 images	**Axial views:** 4CV	✓	✓	X	X	**HIGH (75.5%)**
Yang 2023^37^	545	800 images	595 images	*77 images*	*73 images*	**CHD test set** 66 images **VSD test set** 57 images	**CHD test set** 57 images **VSD test set** 54 images	**Axial views:** 4CV, LVOT, RVOT, 3VV, 3VT	✓	X	X	X	**LOW (40.8%)**
Day 2024^38^	173	NR	NR	NR	NR	NR	NR	**Axial views:** 4CV	X	✓	X	X	**LOW (48.9%)**
Yang 2024^39^	3.850	5.407 images	X	1.797 images	X	1.840 images	508 images	E, A, V waves in the LV in- and out-flow tract	✓	X	X	✓ PWD	**LOW (33.3%)**
Taksoee-Vester 2024^40^	7.373	NR	NR	NR	NR	NR	NR	**Axial views:** Situs, 4CV, LVOT, RVOT, 3VV, 3VT, septum view **Sagittal view:** Ao arch	✓	✓	✓	X	**HIGH (78.4%)**
Zhou 2024^41^	275	96 images	126 images	X	X	21 images	35 images	**Axial views:** 4CV, LVOT, RVOT, 3VV/3VT **Sagittal view:** Ao arch	X	✓	✓	✓ PWD, CD	**MODERATE (51.0%)**

Images are defined as the source used for data collection, while frames or images extracted from videos after data collection, if used, are mentioned in the table's section mentioning the amount of data used for training, validation and testing. Ao, aortic; CD, Color Doppler; CHD, congenital heart defect; FE, fetal echocardiography; LVOT, left ventricular outflow tract; LV, left ventricle; NR, not reported; PWD, pulsed-wave doppler; RVOT, right ventricular outflow tract; VSD, ventricular septal defect; 4CV, four-chamber view; 3VV, three-vessel view; 3VT, three-vessel-trachea.

a Gong et al. used also video screening test dataset 1 and 2, accounting for 51,542 and 67,000 video frames, respectively, for further annotation of videos and data augmentation. In the Table is reported only the amount of data used to test for CHD classification.

B-mode images or still frames extracted from videos were sources of datasets in ten out of fifteen studies.^27^^,^^28^^,^^30^^,^^32^^,^^34^, ^35^, ^36^, ^37^, ^38^, ^39^, ^40^, ^41^ Training with cine-sweeps from the situs to 3VT was used by two studies, with abnormal sweeps used only for testing, but not for training.^29^^,^^31^ Three studies did not mention if still or cine records were used for training, validation and testing.^33^^,^^38^^,^^40^ The median number of participants included to train, validate and test AI models was 341 (interquartile range [IQR] 160–4.399) with a median of total records (images, still frames extracted from videos and videoclips) of 2.687 (IQR 674–10.074). Five studies investigated the implementation of automatic cardiac biometry,^28^^,^^32^^,^^33^^,^^40^^,^^41^ while Doppler data were used by four studies, among which three used pulsed-wave Doppler^32^^,^^39^^,^^41^ and two colour-Doppler.^32^^,^^41^

Pooled proportions showed that, overall, 75% (95% CI 58–88%, I^2^ = 99.9%) of data were used for training, 8% (95% CI 4–12%, I^2^ = 99.4%) for validation and 20% (95% CI 8–37%, I^2^ = 99.9%) for testing AI models (Table 4, Supplementary Figures S1–S6). The most prominent source of heterogeneity between studies was the type of cardiac views collected (Supplementary Table S2).

**Table 4:** Summary of dataset distribution and heterogeneity.

Dataset	Case type	Median (IQR)	Pooled proportions (95% CI)	I^2^ (%)
Training	Normal	800 (292–5.019)	51% (23%–79%)	99.9%
	Abnormal	703 (230–3.104)	27% (10%–49%)	99.9%
Validation	Normal	138 (9–894)	5% (2%–10%)	99.6%
	Abnormal	73 (8–290)	3% (2%–4%)	95.2%
Testing	Normal	102 (24–462)	7% (3%–11%)	99.5%
	Abnormal	150 (31–319)	6% (4%–8%)	97.9%

### AI model performance and comparison with clinicians

The AI model performance, evaluated in retrospect, was compared to clinicians' detection in eleven studies,^27^^,^^28^^,^^30^, ^31^, ^32^^,^^34^, ^35^, ^36^^,^^38^^,^^39^^,^^41^ of which one used a real-world screening programme as a comparator,^36^ three studies used images already annotated or labelled by clinicians and nine studies compared AI vs clinicians’ performance on a specific dataset derived from collected data (Supplementary Table S3). The human comparator group was generally small, with a median of six clinicians (IQR 3–10), and unblinded, meaning that clinicians were aware of the task and expected finding CHDs at higher proportions than those normally seen in routine practice. A median of three clinicians were expert cardiologists (IQR 2–8) and a median of seven were reported as sonographers, trainees or fellows (IQR 4–16).

A relevant source of heterogeneity was the dissimilar prevalence of cardiac abnormalities across different datasets, ranging from 0.9% (in line with a community-screening level prevalence of CHDs) to 30–60% (similar to the frequency seen in a referral population to a fetal cardiology unit).

**Fig. 2:**
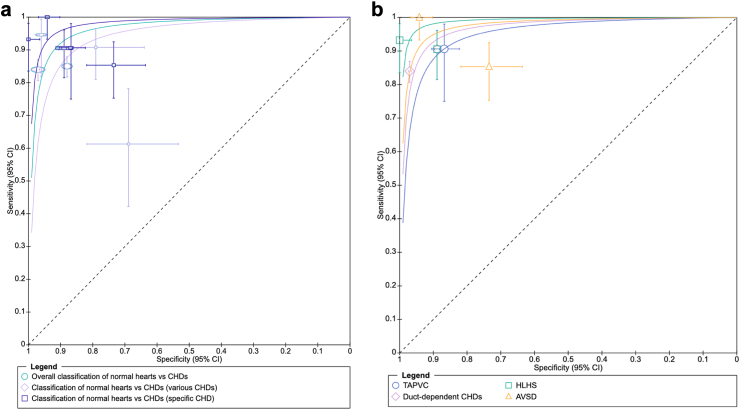
**a (left) shows sROCs for AI classification of normal vs abnormal hearts, when tested with various CHDs or a specific heart defect. b (right) shows sROCs for AI assessment of individual cardiac defects.** CHDs, congenital heart defect; TAPVC, total anomalous pulmonary venous connection; HLHS, hypoplastic left heart syndrome; AVSD, atrioventricular septal defect.

For the binary classification into normal vs abnormal heart, the pooled sensitivity and specificity were 0.89 (95% CI 0.83–0.93, I^2^ 77.92%) and 0.91 (95% CI 0.84–0.95, I^2^ 77.92%) (Fig. 2a, Supplementary Figure S7a and b). For studies assessing AI models trained and tested with normal hearts and all CHDs pooled, the AUC varied from 0.79^29^ to 0.99^28^ and the pooled sensitivity and specificity were 0.85 (95% CI 0.75–0.91, I^2^ 76.27%) and 0.90 (95% CI 0.79–0.96, I^2^ 76.27%), respectively (Fig. 2a, Supplementary Figure S8a and b). The lowest sensitivity and specificity were obtained by the AI model trained with the least number of records, as expected, while the best accuracy metrics were found with the highest number of cases collected.^28^^,^^30^ The leave-one-out sensitivity analysis and exclusion of studies with high risk of bias and low adherence to TRIPOD+AI are summarised in Table 5. The overall estimates remained stable across all iterations, with overlapping confidence intervals, suggesting the robustness of pooled estimates.

**Table 5:** Leave-one-out, exclusion of studies with high risk of bias and low adherence to TRIPOD+AI sensitivity analyses.

Analysis	Excluded study/studies	Pooled sensitivity (95% CI)	Pooled specificity (95% CI)	I^2^
Overall pooled effect size	None	0.89 (95% CI 0.83–0.93)	0.91 (95% CI 0.84–0.95)	77.92%
Leave-one-out analysis				
	Arnaout 2021	0.88 (95% CI 0.82–0.93)	0.90 (95% CI 0.81–0.95)	79.20%
	Nurmaini 2022	0.90 (95% CI 0.85–0.94)	0.92 (95% CI 0.86–0.99)	74.22%
	Truong 2022	0.90 (95% CI 0.83–0.94)	0.91 (95% CI 0.83–0.96)	77.56%
	Wang 2022	0.89 (95% CI 0.82–0.94)	0.91 (95% CI 0.83–0.96)	78.86%
	Day 2023	0.89 (95% CI 0.81–0.93)	0.89 (95% CI 0.82–0.94)	79.40%
	Athalye 2023	0.89 (95% CI 0.82–0.94)	0.92 (95% CI 0.84–0.96)	79.72%
	Tang 2023	0.90 (95% CI 0.83–0.94)	0.89 (95% CI 0.82–0.94)	63.30%
	Zhou 2024	0.87 (95% CI 0.81–0.91)	0.91 (95% CI 0.82–0.96)	73.75%
	Day 2024	0.90 (95% CI 0.82–0.94)	0.92 (95% CI 0.85–0.96)	77.56%
	Taksoee-Vester 2024	0.89 (95% CI 0.82–0.94)	0.91 (95% CI 0.83–0.96)	78.00%
Exclusion of high risk of bias	Nurmaini 2022, Truong 2002, Day 2024	0.92 (95% CI 0.87–0.95)	0.94 (95% CI 0.88–0.97)	70.53%
Exclusion of low adherence to TRIPOD+AI	Nurmaini 2022, Day 2023	0.91 (95% CI 0.86–0.94)	0.93 (95% CI 0.88–0.96)	79.20%

We also assessed models that were tested on a specific cardiac abnormality; there were five of such studies reporting accuracy metrics, and these aimed to detect the total anomalous pulmonary venous connection, hypoplastic left heart syndrome, atrioventricular septal defects and coarctation of the aorta.^32^^,^^36^^,^^38^^,^^40^^,^^41^ Pooling these data showed that the reported AI models achieved an overall sensitivity and specificity of 0.92 (95% CI 0.87–0.96, I^2^ 0.04%) and 0.91 (95% CI 0.81–0.97, I^2^ 0.04%) (Fig. 2a, Supplementary Figure S9a and 9b). In more detail, one study developed an algorithm for detecting hypoplastic left heart syndrome that, in a per-fetus analysis, reached a sensitivity and specificity of 100% and 94%, respectively.^36^ Models developed to screen atrioventricular septal defect achieved a sensitivity and specificity of 86.8–100% and 72.8–94.1%.^38^^,^^41^ Conversely, for coarctation of the aorta, the reported sensitivity and specificity were 90.4% and 88.9%.^40^ Lastly, total abnormal pulmonary vein returns were detected by the AI model with a sensitivity and specificity of 94.7% and 81.7%, respectively^32^ (Fig. 2b, Supplementary Table S3).

Overall, studies stated that AI models outperformed less expert operators (such as fellows, junior sonographers, residents and trainees), but were less accurate than experts in fetal cardiology. Only two studies showed that clinicians’ performance improved if AI-assisted with binary outputs (normal vs abnormal).

### Risk of bias, adherence to reporting standards and certainty of evidence

**Fig. 3:**
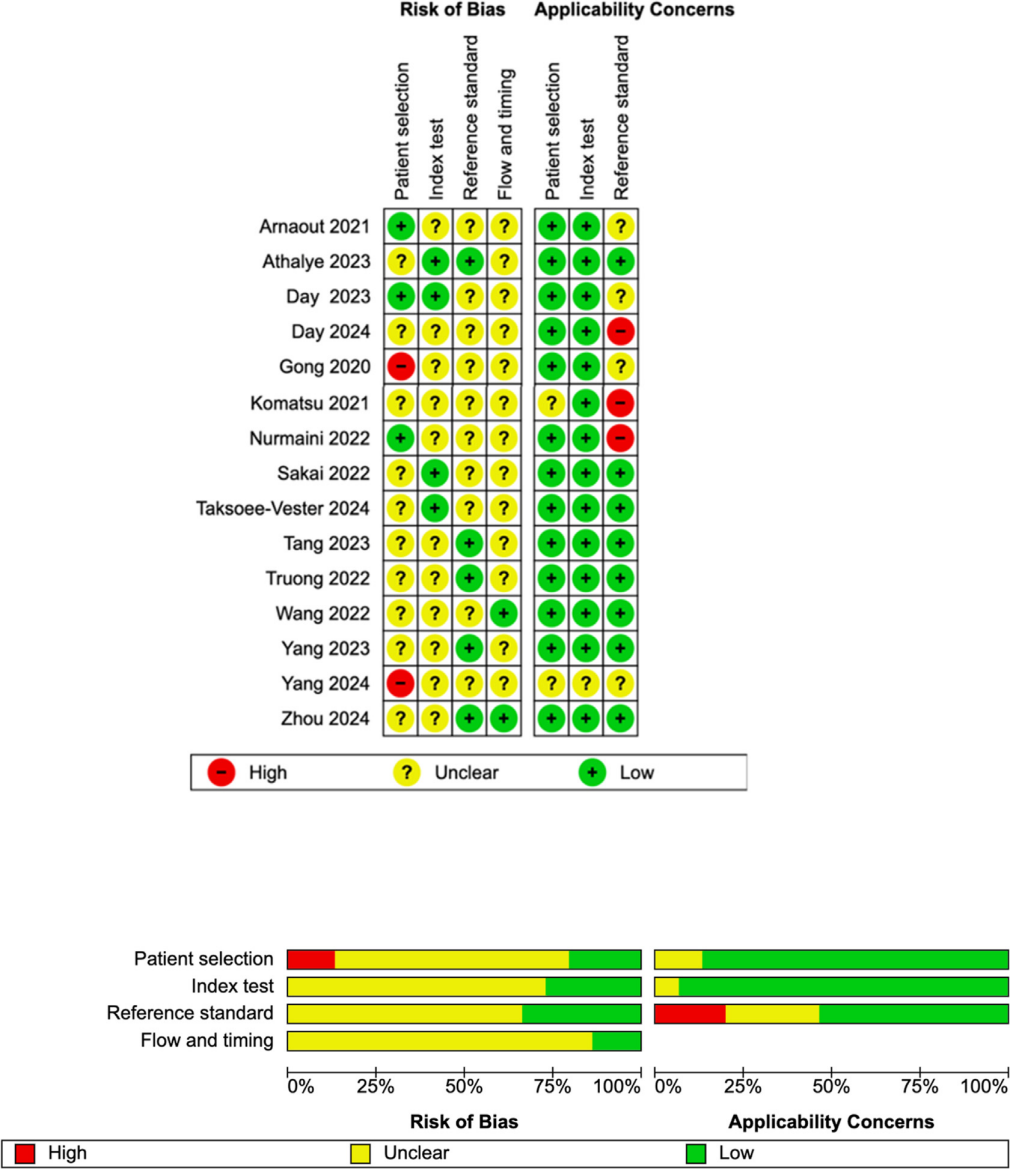
**QUADAS-2 assessment**.^25^

The overall risk of bias using QUADAS-2 classified three studies as high-risk in the domain of applicability of the reference standard, and two studies for patient selection (Fig. 3). The remaining studies were of unclear-risk, mostly for patient selection, the conduct or the interpretation of the index test and reference standard, and the flow of data.

**Fig. 4:**
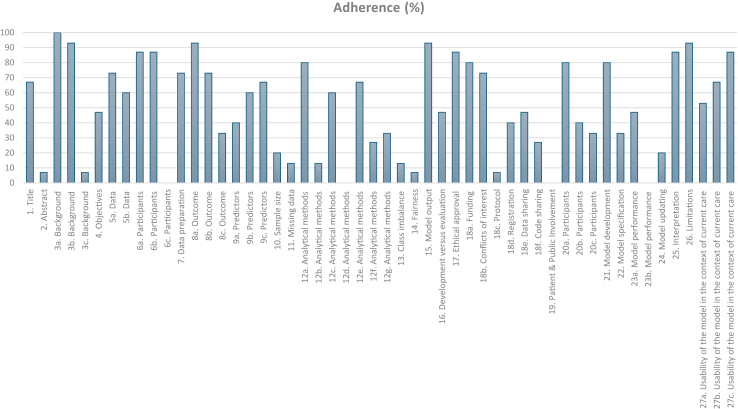
**TRIPOD+AI assessment,** adapted from TRIPOD+AI 2024 checklist.^19^

For reporting standards, on a per-item analysis, optimal adherence (>70%) was present for 35% TRIPOD+AI items (18/51) (Fig. 4). Overall, the included studies adhered to TRIPOD+AI items with a median of 53% (IQR 27–80%). On a per-study analysis, three studies were rated as highly adherent, six as moderately, and six as low (Supplementary Table S3). All the included studies were submitted for publication before the TRIPOD+AI checklist was released. However, only two studies explicitly stated their intention to comply with the reporting standards available at the time of publication, as TRIPOD.^40^, ^41^, ^42^

Publication bias was evaluated using Deek's test, suggesting an absence of statistically significant publication bias in this analysis (p = 0.474). The coefficient for invess was −6.20 (95% CI, −32.25 to 19.86; p = 0.582), indicating no significant relationship between study size and effect estimates. Overall, these findings do not support the presence of small-study effects, although the limited number of the included studies may reduce the statistical power to detect subtle biases.

Summary of findings applying GRADE showed low level of evidence for the primary outcome and subgroup analyses (Supplementary Table S4).

## Discussion

This systematic review and meta-analysis thoroughly analysed the currently available literature on AI applied to the prenatal detection of heart defects, aligning with recent standards, as TRIPOD+AI.^19^ AI model performance for the classification of normal vs abnormal heart achieved a high pooled sensitivity and specificity with a higher pooled sensitivity when tested with specific cardiac defects rather than various CHDs, but a similar specificity. Compared to clinicians, AI models performed better than less experienced operators, but not as accurately as experts in the reported studies.

These results illustrated the emergent attempts to implement AI to support decision-making in fetal cardiology. In fact, over the last five years, the interest has progressively moved from the recognition of cardiac planes in normal fetuses, towards the classification of CHDs, and the evaluation of diagnostic performance. Initial studies showed that, amongst all anatomical views, cardiac views were the hardest to recognise, due to the dynamicity of cardiac cycles and the “category confusion” produced by different scanning angles,^43^ while, more recently, AI was found to correctly classify cardiac planes in over 90% of cases, with overall good agreement with experts.^18^ Automated segmentation has shown potential in the automated analysis of cardiac morphology, and may aid in obtaining automated measurements, such as of the cardiac axis or ventricular diameters.^28^^,^^40^ These efforts in AI-based analysis of normal cardiac anatomy have laid the groundwork for abnormality detection as a recent development—with all published studies in this review after 2020.

So far, AI fetal cardiac examination have been evaluated mainly through models trained and tested with images or still frames extracted from videos, while only two studies used video-analysis.^29^^,^^31^ Although there is no consensus on whether videos are superior to still images, or vice versa, for fetal heart evaluation, in clinical practice an operator sweeps through the heart to evaluate the spatial relationships of the cardiac structures using moving images. In our opinion, the strength of ultrasound as a real-time imaging modality is somewhat neglected when evaluating still images only, and this is of particular significance when it comes to the rapidly beating fetal heart. This real-time element also suggests that integration of AI models with acquisition is the most likely mode to work in practice, with AI-based-software embedded in ultrasound machines.^29^^,^^31^^,^^44^

An established way to improve the recognition of structural cardiac defects is the application of colour-Doppler.^7^^,^^45^ Notably, only two included studies used a protocol for AI model development and evaluation with colour-Doppler images, and it is still unclear whether AI models may perform better if trained with colour-Doppler data, or whether this may in fact increase category confusion.

Although not routinely recommended, cardiac measurements were previously found to improve prenatal detection and prognostic assessment of cardiac defects, such as coarctation of the aorta, hypoplastic left heart syndrome and atrioventricular septal defects.^46^^,^^47^ Manual measurement of cardiac biometry is a time-consuming task that is mainly performed in targeted examination by experts in fetal cardiology, but not in routine screening. Automation of these measurements might expedite this process and assist in their acquisition. Five studies explored the automated cardiac biometry, with one showing the feasibility of implementing this to improve early prediction of aortic coarctation.^40^ This is a critical heart defect that is subtle, and often missed prenatally,^48^ so further studies in this direction are warranted.

The majority of studies conclude that AI outperforms less experienced sonographers, achieving diagnostic accuracy that approaches expert levels. AI assistance may also enhance performance, particularly for operators with lower expertise. However, these findings must be interpreted cautiously, as expertise in fetal cardiology remains inconsistently defined. Although some studies have attempted to define expertise based on years of experience in fetal cardiac US–ranging from more than 10 years to 15–25 years -^34^^,^^35^^,^^39^(Supplementary Table S2), for which a threshold of at least 10 years might be considered a reasonable benchmark, others rely on case volume, participation in structured training, or professional titles without specifying whether these pertain to clinical or ultrasonographic skills. How clinical experience translates to ultrasonographic skill is also a matter that current studies inadequately address, despite its relevance, considering that variability in US data acquisition can introduce significant data heterogeneity. A further issue concerns the false positive and false negative rates reported in experienced hands, up to 5%.^28^ These rates in high-expertise units are difficult to establish a-priori, being highly dependent on dataset composition and case complexity (coarctartion of the aorta,^28^^,^^34^ total abnormal pulmonary venous returns,^28^^,^^34^ anomalous left coronary artery from the pulmonary artery,^34^ aortopulmonary window,^34^ Supplementary Table S1). Given these challenges, future research should prioritize the development of clear and standardized criteria for defining expertise in fetal cardiology, across varying levels of case complexity, to improve the reliability of AI-assisted diagnostic approaches in fetal cardiology. Despite encouraging sensitivity, AI models dealing with detection of rare CHDs, less likely to be diagnosed prenatally, performed at the expense of a lower specificity. In practice, use of such models would result in large increases of false-positive referrals to fetal cardiology units.^49^ Moreover, the results should be considered in light of the overall unclear-to high-risk of bias, sub-optimal adherence to reporting standards, and low GRADE certainty, although we acknowledge that the included studies are at an earlier point of diagnostic accuracy study developing and testing. Significant biases arose in most studies from the selection of patients, the conduct of the index test and reference standard, and the flow of data. It is unclear whether patient selection might have been conducted leading to better AI performance. In line with this risk, some studies used only high-quality images to develop AI models, which might introduce bias and reduce applicability in a real clinical scenario where low-quality images may be obtained. Moreover, three studies considered the clinical interpretation of cardiac findings as a reference standard, without postnatal confirmation, meaning there is uncertainty if only true positives were detected by cardiologists prenatally. Lastly, among multicentre studies, the process of handling data clustering was not clearly reported,^50^ and together with the limited external validation, it challenges the estimation of AI real-world performance, considering the variations in ultrasound machines and imaging protocols, all of which may impact AI models’ performance. Future studies should prioritize external validation to assess AI performance across different populations, clinical settings, and healthcare infrastructures, as the use of quality control and the adherence to standardized imaging protocols and international guidelines, to minimize variability introduced by different machines and operator techniques. Open sources without data sharing constraints are also needed to maximize generalizability. Until this is done, confidence in applying these AI models broadly remains limited. These aspects should be carefully considered when examining the thin line between progress in fetal cardiology and potential harms. The emerging challenge of “trust calibration”, referring to the ability of clinicians to rely on correct AI outputs while overriding them when erroneous to prevent misdiagnoses, is key in this context. How this might be appropriately achieved is still a matter of ongoing debate. Moreover, the AI model may be correct when the human is wrong, and even less is understood about the best way to deal with this scenario.^38^ Explainable AI (XAI) techniques, such as saliency maps, attention mechanisms, or visual explanation methods, have the potential to address clinical concerns by illustrating how AI systems reach decisions. Although it has been suggested that incorporating XAI can enhance clinician trust in AI predictions, XAI remains underexplored in prenatal cardiac ultrasound, and future research should prioritise evaluating explainable methods in terms of transparency, interpretability, and clinical acceptability.^32^

The strength of our review is the systematic and detailed assessment of AI performance for CHD detection and characterization. We sought to understand the clinical potential for such models in the context of prenatal screening, rather than a technical perspective on the feasibility of implementation. We also acknowledge the limitations of this systematic review, mostly arising from limitations in the constituent studies. Firstly, the included studies mainly conducted retrospective data collection, and it is possible that prospective evaluation may give a different result on AI performance. In fact, we hypothesise that real-world AI model performance may well be weaker, due to a higher proportion of lower-quality records and operator-dependent factors in clinical practice. Second, we have identified prominent sources of heterogeneity, from the heart views collected to the dissimilar prevalence and types of CHDs across different datasets. Lastly, the results of this systematic review are mostly from HICs and tertiary level units, where implementation of AI is expected to be of less relevance compared to LMICs or community-screening levels.

Most studies into AI prenatal detection of CHD have moderate-high risk of bias with sub-optimal adherence as assessed against the TRIPOD+AI reporting standards. Heterogeneity applies to most studies in terms of datasets, protocols used for model development and testing, healthcare settings and types of heart defects considered. The conclusion that the performance of AI models is better than non-expert clinicians, and slightly inferior to experts in fetal cardiology, should be interpreted with caution. Limitations exist due to the relatively small number of studies, affecting power in detecting publication bias and influencing GRADE certainty. We call on researchers for future studies to follow reporting standards to reduce the risk of bias and sources of heterogeneity. Furthermore, there is a lack of prospective studies conducted at community-screening level, where implementation of AI-based systems might be of greatest benefit. Alongside technical developments in the fields of colour-Doppler and cardiac biometry, future prospective studies of diagnostic accuracy are needed.

## Contributors

ATP, JAN: Funding acquisition, project administration; ED, OP, ATP: Conceptualisation; EB, OP, ATP: Design of methodology; ED, OP, CS: Literature search; ED, OP: Data analysis; ED, OP, ATP, JAN, NHC, BS: Data interpretation; EB, OP, ATP: Writing–original draft; All authors: Writing—review & editing: all authors read and approved the final version of the manuscript. ATP, ED and OP have directly accessed and verified the underlying data reported in the manuscript.

## Data sharing statement

Data in this systematic review are available in the individual included articles. Data supporting the findings are available from the corresponding author upon reasonable request.

## Declaration of interests

ATP and JAN are senior advisors for Intelligent Ultrasound, undertaken via Oxford University Innovations which manages the consulting activities of University staff. All other authors declare no competing interests.

## Appendix A Supplementary data

Supplementary Tables and Figures **Supplementary Figure 1. Training with normal fetal heart data.** Forest plot showing the proportion of normal fetal heart data used for training across included studies. **Supplementary Figure 2. Training with abnormal fetal heart data.** Forest plot showing the proportion of abnormal fetal heart data used for training across included studies. **Supplementary Figure 3. Validation with normal fetal heart data.** Forest plot showing the proportion of normal fetal heart data used for validation across included studies. **Supplementary Figure 4. Validation with abnormal fetal heart data.** Forest plot showing the proportion of abnormal fetal heart data used for validation across included studies. **Supplementary Figure 5. Testing with normal fetal heart data.** Forest plot showing the proportion of normal fetal heart data used for testing across included studies. **Supplementary Figure 6. Testing with abnormal fetal heart data.** Forest plot showing the proportion of abnormal fetal heart data used for testing across included studies. **Supplementary Figure 7. a Forest plot and overall pooled specificity and sensitivity for AI classification of normal vs abnormal hearts.** CHDs, congenital heart defects; TAPVC, total anomalous pulmonary venous connection; HLHS, hypoplastic left heart syndrome; AVSD, atrioventricular septal defect; CoA, coarctation of the aorta. **b sROC for overall AI classification of normal vs abnormal hearts** **Supplementary Figure 8. a Forest plot and pooled specificity and sensitivity for AI classification of normal vs abnormal hearts (model tested with various CHDs). b sROC for AI classification of normal vs abnormal hearts (model tested with various CHDs).Supplementary Figure 9. a Forest plot and pooled specificity and sensitivity for AI classification of normal vs abnormal hearts (model tested with a specific cardiac abnormality)** TAPVC, total anomalous pulmonary venous connection; HLHS, hypoplastic left heart syndrome; AVSD, atrioventricular septal defect; CoA, coarctation of the aorta. **b sROC for AI classification of normal vs abnormal hearts (model tested with a specific cardiac abnormality)**.

Appendix

2X2 tables
