# Supplementary material for: Artificial intelligence-enabled prenatal ultrasound for the detection of fetal cardiac abnormalities: a systematic review and meta-analysis
Source: eClinicalMedicine. 2025 May 30;84:103250. doi: 10.1016/j.eclinm.2025.103250 (PMC12273734; doi:10.1016/j.eclinm.2025.103250)

**Supplementary Table 1. Cardiac defects collected in the included studies**

| **Study**  **(year)** | **Type of CHDs** |
| --- | --- |
| **Gong**  **2020^(27)^** | Abnormal cardiothoracic proportion, abnormal number of heart chambers, abnormal atrioventricular septum, abnormal atrioventricular connection, mitral valve disease/tricuspid valve disease, abnormal ratio of heart chambers, cardiac cavity occupation, and abnormal blood vessels |
| **Arnout**  **2021^(28)^** | ToF, PAIVS, PAVSD DORV, HLHS, isolated aortic stenosis, AVSD, TGA, single ventricle, including heterotaxy with left or right atrial isomerism, DORV, TAPVR; truncus arteriosus; Ebstein anomaly; tricuspid atresia. |
| **Komatsu**  **2021^(29)^** | AVSD, Tricuspid atresia, ToF, DORV,HLHS, Ebstein, TGA, TOF, Tricuspid atresia, persistent right fourth aortic arch, PAIVS |
| **Nurmaini**  **2022^(30)^** | ASD, VSD, AVSD, Ebstein Anomaly, TOF, TGA, HLHS |
| **Sakai**  **2022^(31)^** | AVSD, DORV-AVSD, CoA-VSD, TGA, Tricuspid atresia, Ebstein anomaly, ToF, DORV-TGA-VSD, PAIVS, RAA, HLHS |
| **Wang**  **2022^(32)^** | TAPVR |
| **Truong**  **2022^(33)^** | AVSD, VSD, HLHS, Ebstein anomaly, pulmonary stenosis, DORV. |
| **Athalye**  **2023^(34)^** | ALCAPA, AoP window, AVSD, CoA, DORV, Double arch, TGA, IAA, PAIVS, pulmonary sling, pulmonary stenosis, right atrial isomerism, Shone complex, TAVPR, ToF, truncus arteriosus, VSD |
| **Tang**  **2023^(35)^** | TGA, IAA, CoA |
| **Day**  **2023^(36)^** | HLHS |
| **Yang**  **2023^(37)^** | VSD, ToF, TGA, DORV, persistent truncus arteriosus, AVSD, persistent left SVC, ARSA, RAA and other defects. |
| **Day**  **2024^(38)^** | AVSD |
| **Yang**  **2024^(39)^** | ST, PAC, AVB |
| **Taksoee‐Vester 2024^(40)^** | CoA |
| **Zhou**  **2024^(41)^** | AVSD, HRHS, VSD, HLHS |

ALCAPA, anomalous left coronary artery from pulmonary artery; ARSA, aberrant right subclavian artery; AVB, atrioventricular block; AVSD, atrioventricular septal defect; CoA, aortic coarctation; DORV double-outlet right ventricle; HLHS, hypoplastic left heart syndrome; HRHS, hypoplastic right heart syndrome; PAC, premature atrial contractions; PAIVS, pulmonary atresia with intact ventricular septum; PAVSD, pulmonary atresia with ventricular septal defect; ST, Sinus tachycardia; TAPVR,total anomalous pulmonary venous return; TGA,transposition of the great arteries; TOF, tetralogy of Fallot; VSD, ventricular septal defect.

**Supplementary Table 2. AI model performance and comparison with current screening programme or clinician performance**

| **Study**  **(year)** | **Clinicians**  **(N)** | **Level of expertise** | **Conclusions** |
| --- | --- | --- | --- |
| **Gong**  **2020^(27)^** | 9 | Expert cardiologists with junior titles  Expert cardiologists with intermediate titles  Expert cardiologists with senior titles | **Dataset prevalence unclear**  DGACNN outperforms doctors with junior and intermediate titles by around 4%-8%, although it is still 2.3% lower than doctors with senior titles in terms of the average accuracy. Average accuracy for the doctors with the three titles is 81%, which is 3% lower than that of DGACNN, 84%, in the test. |
| **Arnout**  **2021^(28)^** | 7 | Fetal cardiology and maternal–fetal medicine attendings  Experienced fetal cardiology sonographers,  Fetal cardiology fellows | **OB-4000 (0.9% mixed CHD)**  The model achieved an area under the curve (AUC) of 0.99, 95% sensitivity (95% CI, 84–99%), 96% specificity (95% CI, 95–97%) and 100% negative predictive value in distinguishing normal from abnormal hearts. Model sensitivity was comparable to that of clinicians and remained robust on outside-hospital and lower-quality images.  **OB-125 (0.9% mixed CHD)**  The model achieved an area under the curve (AUC) of 0.93, 88% sensitivity (95% CI, 47–100%) and 90% specificity (95% CI, 73–98%). Clinicians achieved an average sensitivity of 86% (95% CI, 82–90%) and a specificity of 68% (95% CI, 64–72%). The model was comparable to clinicians (P = 0.3) in sensitivity and superior to them (P = 0.04) in specificity. |
| **Komatsu**  **2021^(29)^** | NA | NA | **Dataset (3.85% mixed CHD)**  The reported AUC was 0.78 and 0.89 in the 4CV and 3VT, respectively.  No comparison with clinicians was reported. |
| **Nurmaini**  **2022^(30)^** | 3 | Expert fetal cardiologists | **Dataset (40.8% mixed CHD)**  The reported AI model’s accuracy, sensitivity and specificity before data augmentation and after inter-patient testing was 71%, 62% and 68%, failing to correctly classify between CHD with overlapping features (i.e. TOF and VSD).  The reported clinicians’ performance was similar to the AI model for one expert cardiologist, while the other two underperformed. |
| **Sakai**  **2022^(31)^** | 21 | Fetal cardiology experts  Fellows  Residents | **Dataset (8.12% mixed CHD)**  The reported AUC for the AI algorithm only was 0.86 (0.858 – 0.865). Experts (n = 8) achieved an AUC of 0.96 (0.95 – 0.99), that increased to 0.98 (0.94-1.0) if AI-assisted. Fellows (n =10) achieved an AUC of 0.85 (0.67 – 0.93), that increased to 0.89 (0.83- 0.95) if AI-assisted. Residents’ (n = 9) performance was 0.63 (0.37 - 0.77) and 0.75 (0.47-0.84) with AI-assistance. |
| **Wang**  **2022^(32)^** | NR | NR | **Dataset (10% TAPVC)**  The AI segmentation models, using PLAS ratio as a cardiac biometric measurement, had an AUC ranging from 0.85 (95% CI 0.74-0.96) at the lowest to 0.94 (95% CI 0.89-0.99). The sensitivity ranged from 79% (95% CI 54.4-93.9) to 94.7% (95% CI 74.0-99.9) and the specificity from 81.7% (95% CI 71.6-89.4) to 86.6% (95% CI 77.3-93.1).  The clinicians reached an AUC of 0.97 (95% CI 0.92-0.99), a sensitivity of 100% (95% CI 82.4%-100%) and a specificity of 86.6% (95% CI 77.3 -93.1). |
| **Truong**  **2022^(33)^** | NA | NA | **Dataset prevalence (14% mixed CHD)**  The AI model provided a sensitivity of 0.85, a specificity of 0.88, a positive predictive value of 0.55 and a negative predictive value of 0.97 with a mean AUC of 0.94.  No comparison with clinicians was reported. |
| **Athalye**  **2023^(34)^** | 3 | Fetal cardiac experts (fetal cardiac US experience ranging from 15 to 25 years) | **Dataset (mixed CHD 60% - the model was not trained for 19/47 lesions showed)**  The AI model’s sensitivity and specificity was 91% and 78% respectively, while blinded clinical experts had a sensitivity of 55% (range, 47-67%) and specificity of 71% (range, 57-83%). Thus, model outperforms clinicians for sensitivity ( p= 0.03), but was similar for specificity. |
| **Tang**  **2023^(35)^** | 6 | Senior sonographers (more than 10 years of experience)  Junior sonographers (5-10 years of experience)  Most-junior sonographers (1-2 years of experience) | **Dataset (8.1% duct-dependent CHDs - IAA, CoA, TGA**)  The AI algorithm had an accuracy of 91.4% for TGA, 74.2% for CoA and 100% for IAA with a sensitivity for duct-dependent CHD of 0.84 (95% CI 0.73 - 0.91) and specificity of 0.97 (95% CI 0.90 - 0.99).  The less experienced operators reached an accuracy of 94.3% for TGA, 51.6% for CoA and 100% for IAA, with a sensitivity and specificity for duct-dependent CHD of 0.74 (95% CI 0.58 - 0.86) and 0.96 (95% CI 0.84 - 0.99).  The most experienced reached an accuracy of 94.3% for TGA, 77.4% for CoA and 100% for IAA, with a sensitivity and specificity for duct-dependent CHD of 0.87 (95% CI 0.76 - 0.94) and 0.86 (95% CI 0.76 - 0.92). |
| **Day**  **2023^(36)^** | X | Real-world screening performance | **Dataset (36.6% HLHS)**  The sensitivity of the UK screening programme was estimated to range from 92.1% to 95.9% and the specificity was found to be 99.9%.  The AI model calibrated for sensitivity (100%, 95% CI 50.9%–100%) had a specificity of 94% (95% CI 60.4%–99.4%), while calibrated for specificity (100%, 95% CI 68.0%–100%) had a sensitivity of 93.3% (95% CI 49.9%–99.8%). In the first scenario, AI could detect 10 cases of HLHS missed by the UK screening programme at the expense of 45,240 false positives, whereas, in the second scenario, the AI might have missed 2 cases of HLHS with no referral. |
| **Yang**  **2023^(37)^** | N/A | N/A | **Dataset (43.78% mixed CHD with overall 3.31% of VSD)**  Data are reported for YOLOv5 that had better performance and shorter identification time than ResNet50 and MobileNetv2  Tested on the CHD dataset, the accuracy in classification normal versus abnormal hearts ranged from 72.36% to 82.93%)  Tested on VSD dataset, the accuracy in recognition of VSDs ranged from 75.93% to 92.9%). |
| **Day**  **2024^(38)^** | 10 | Consultant fetal cardiologists  Trainee pediatric cardiologists  Fetal cardiac sonographers | **Dataset (43.3% AVSD)**  The AI model alone achieved an accuracy of 0.79 (95% CI 0.76 – 0.83), sensitivity of 0.86 (95% CI 0.83 – 0.90), specificity 0.73 (95% CI 0.70 - 0.75).  Consultant fetal cardiologists (n = 2) achieved an accuracy of 0.89 (95% CI 0.87 – 0.91). Trainee pediatric cardiologists (n =3) achieved an accuracy of 0.77 (95% CI 0.75 – 0.79). Fetal cardiac sonographers’ (n = 5) performance was 0.86 (95% CI 0.85 - 0.88). When AI-assisted, clinicians’ performance slightly increased with binary AI output (normal vs abnormal), but not with temperature scale model or grad-CAM assistance. |
| **Yang**  **2024^(39)^** | 2 | Senior expert cardiologists (more than 14 years of experience) | **Dataset (5.32% of fetal arrhythmias – TS, PAC, AVB)**  The proposed model had a sensitivity of 95%, 96.67% and 96.15% in recognizing PAC, AVB and TS.  The Kappa coefficient is 93.74%, indicating that intelligent diagnosis of HOCAD is highly consistent with cardiologists’ diagnosis. |
| **Taksoee‐Vester 2024^(40)^** | NA | NA | **Dataset (1% CoA)**  The best predictive performance was reached including six cardiac biometrics (RV_a_, LV_a_, Dao_d_, Ao_d_, MPA_d_, LA_a_) with an AUC of 0.96, a sensitivity of 90.4% (95% CI 83.7-97.2) and a specificity of 88.9% (95% CI 88.2 – 89.6%).  No comparison with clinicians was reported. |
| **Zhou**  **2024^(41)^** | NR | NR | **Dataset (57.9% mixed CHD with 20.1% of AVSD)**  The AUC acquired from the AI models could reach 0.99 (CI 95% 0.96–1.00), which was the same as the expert labelled. The sensitivity and specificity were 1.00 (CI 95% 0.59–1.00) and 0.94 (CI 95% 0.71–0.99), respectively. |

**Supplementary Table 3. Excluded studies**

| **Study** | **Title** | **Reason for exclusion** |
| --- | --- | --- |
| **Han et al. 2022** | Adoption of Compound Echocardiography under Artificial Intelligence Algorithm in Fetal Congenial Heart Disease Screening during Gestation | Retracted article. |
| **Barber et al. 2024** | Advances in Fetal Cardiac Imaging and Intervention | Review article. |
| **Zhang et al. 2024** | Advances in the Application of Artificial Intelligence in Fetal Echocardiography | Review article. |
| **Femina et al.2019** | Anatomical structure segmentation from early fetal ultrasound sequences using global pollination CAT swarm optimizer-based Chan-Vese model | No CHDs in the dataset. |
| **Wu et al. 2023** | Application of Artificial Intelligence in Anatomical Structure Recognition of Standard Section of Fetal Heart | No CHDs in the dataset. |
| **Su et al. 2022** | Application of computer-aided diagnosis of congenital heart disease in four-chamber view of fetal heart basic screening | Editorial correspondence. |
| **Cai et al. 2022** | The Application of Knowledge Distillation toward Fine-Grained Segmentation for Three-Vessel View of Fetal Heart Ultrasound Images | No CHDs in the dataset. |
| **Liu et al. 2024** | Applications of artificial intelligence-powered prenatal diagnosis for congenital heart disease | Review article. |
| **Temsah et al. 2024** | Art or Artifact: Evaluating the Accuracy, Appeal, and Educational Value of AI-Generated Imagery in DALL.E 3 for Illustrating Congenital Heart Diseases | Postnatal evaluation of CHDs. |
| **Steinhard et al. 2022** | Artificial intelligence and simulation in prenatal medicine-What we can learn from machines | Review article. |
| **Enache et al. 2024** | Artificial Intelligence in Obstetric Anomaly Scan: Heart and Brain | Review article. |
| **Reddy et al. 2022** | Artificial intelligence in perinatal diagnosis and management of congenital heart disease | Review article. |
| **Weichert et al. 2022** | Artificial intelligence in prenatal cardiac diagnostics | Review article. |
| **Day et al. 2021** | Artificial intelligence, fetal echocardiography, and congenital heart disease | Review article. |
| **Wang et al. 2021** | Automated interpretation of congenital heart disease from multi-view echocardiograms | Postnatal evaluation of CHDs. |
| **Ling et al. 2024** | Automatic segmentation of 15 critical anatomical labels and measurements of cardiac axis and cardiothoracic ratio in fetal four chambers using nnU-NetV2 | No CHDs in the dataset. |
| **Bahado-Singh et al. 2023** | Cell-Free DNA in Maternal Blood and Artificial Intelligence: Accurate Prenatal Detection of Fetal Congenital Heart Defects | AI not applied to US recognition of CHDs. |
| **Yan et al.2024** | A deep learning framework for identifying and segmenting three vessels in fetal heart ultrasound images | Classification between normal vs abnormal hearts was not assessed. |
| **Ai et al. 2021** | Detection of ventricular septal areas in fetal cardiac ultrasound videos utilizing deep learning | Conference abstract. |
| **Xu et al. 2020** | DW-Net: A cascaded convolutional neural network for apical four-chamber view segmentation in fetal echocardiography | Classification between normal vs abnormal hearts was not assessed. |
| **Masaaki et al. 2021** | Explainable artificial intelligence to support examiners for abnormality detection in fetal cardiac ultrasound screening | Conference abstract. |
| **Drukker 2024** | The Holy Grail of obstetric ultrasound: can artificial intelligence detect hard-to-identify fetal cardiac anomalies? | Editorial article. |
| **Dozen et al. 2020** | Image segmentation of the ventricular septum in fetal cardiac ultrasound videos based on deep learning using time-series information | Classification between normal vs abnormal hearts was not assessed. |
| **Reddy et al. 2022** | Improving Prenatal Detection of Heart Disease Scalable Composite Analysis of Six Fetal Cardiac Biometrics | Conference abstract. |
| **Zhu et al. 2023** | Improving the Quality of Fetal Heart Ultrasound Imaging With Multihead Enhanced Self-Attention and Contrastive Learning | Classification between normal vs abnormal hearts was not assessed. |
| **Garcia Canadilla et al. 2020** | Machine Learning in Fetal Cardiology: What to Expect | Review article. |
| **Stos et al. 2024** | Overriding aorta identification using artificial intelligence applied to second-trimester fetal ultrasound | Conference abstract. |
| **Sriraam et al. 2023** | Performance evaluation of computer-aided automated master frame selection techniques for fetal echocardiography | No CHDs in the dataset. |
| **Velly et al. 2022** | Postnatal diagnosis of congenital heart defects in Southeast of France: An audit of prenatal ultrasonographic practices and can we improve fetal screening by adding AI (artificial intelligence) solutions? | Conference abstract. |
| **Qiao et al. 2023** | A Pseudo-Siamese Feature Fusion Generative Adversarial Network for Synthesizing High-Quality Fetal Four-Chamber Views | Classification between normal vs abnormal hearts was not assessed. |
| **Lu et al. 2022** | A YOLOX-based Deep Instance Segmentation Neural Network for Cardiac Anatomical Structures in Fetal Ultrasound Images | Classification between normal vs abnormal hearts was not assessed. |
| **Habib et al. 2024** | 95 Application of machine learning in the detection of four chamber abnormalities | Conference abstract. |
| **Nurmaini et al. 2021** | Deep Learning-Based Computer-Aided Fetal Echocardiography: Application to Heart Standard View Segmentation for Congenital Heart Defects Detection. | Same outcomes and possibly study population as Nurmaini et al. 2022, included in the final analysis. |
| **Nurmaini et al. 2023** | Automatic echocardiographic anomalies interpretation using a stacked residual-dense network model | Datasets enriched with both prenatal and postnatal CHDs. |
| **An et al. 2021** | A category attention instance segmentation network for four cardiac chambers segmentation in fetal echocardiography | Classification between normal vs abnormal hearts was not assessed. |
| **An et al. 2021** | Fetal Heart and Descending Aorta Detection in Four-Chamber View of Fetal Echocardiography | Classification between normal vs abnormal hearts was not assessed. |
| **Ling et al.2024** | A Coarse-Fine Collaborative Learning Model for Three Vessel Segmentation in Fetal Cardiac Ultrasound Images | Classification between normal vs abnormal hearts was not assessed. |
| **Pu et al. 2024** | HFSCCD: A Hybrid Neural Network for Fetal Standard Cardiac Cycle Detection in Ultrasound Videos | Classification between normal vs abnormal hearts was not assessed. |
| **Yang et al. 2024** | An intelligent quantification system for fetal heart rhythm assessment: A multicenter prospective study | Same outcomes and possibly study population as Yang et al. 2024, included in the final analysis. |
| **An et al. 2021** | Simultaneous Segmentation of Four Cardiac Chambers in Fetal Echocardiography | Classification between normal vs abnormal hearts was not assessed. |
| **Pietrolucci et al. 2023** | Evaluation of an artificial intelligent algorithm (Heartassist TM) to automatically assess the quality of second trimester cardiac views: a prospective study | Classification between normal vs abnormal hearts was not assessed. |
| **Arunamata et al. 2023** | Evaluation of a Deep Neural Network for Detection of D-Transposition of the Great Arteries on Fetal Echocardiograms | Conference abstract. |
| **Yu et al. 2024** | Deep learning-based differentiation of ventricular septal defect from tetralogy of Fallot in fetal echocardiography images | Only abnormal cases collected. |
| **Lu et al. 2024** | SKGC: A General Semantic-Level Knowledge Guided Classification Framework for Fetal Congenital Heart Disease | Classification between normal and abnormal fetal heart was not assessed. The authors mentioned the model’s performance in classifying between normal and abnormal 4CVs (NA-4CH), but it is unclear whether abnormal 4CVs actually corresponded to abnormal fetal heart (eg, physiological ventricular disproportion in the third trimester) while no abnormal views were reported in FEST dataset. |
| **Li et al. 2024** | Application of artificial intelligence in VSD prenatal diagnosis from fetal heart ultrasound images | Inclusion of fetal US < 16 weeks (lower gestational age reported was 14 weeks). |
| **Taksøe-Vester et al. 2025** | Correction to 'Role of artificial-intelligence-assisted automated cardiac biometrics in prenatal screening for coarctation of aorta' | Data included in our meta-analysis were not edited in this erratum. |
| **Aoyama et al.**  **2024** | Automated Assessment of the Pulmonary Artery-to-Ascending Aorta Ratio in Fetal Cardiac Ultrasound Screening Using Artificial Intelligence. | Same study population as Komatsu et al. 2021, included in the final analysis |

# Supplementary Table S4. Summary of findings - GRADE Assessment for AI Diagnostic Accuracy

| **Domain** | **Overall Assessment**  **(n = 10)** | **Detection in model reading specific CHDs (n=5)** | **Detection in model reading multiple CHDs (n = 5)** |
| --- | --- | --- | --- |
| **Risk of Bias** | 3/10 studies with high risk of bias (QUADAS-2)  Unclear risk in the remaining studies | 1/5 studies with high risk of bias (QUADAS-2)    Unclear risk in the remaining studies | 1/5 studies with high risk of bias (QUADAS-2)  Unclear risk in the remaining studies |
| **Inconsistency** | High heterogeneity  (I² = 77.92%)  Sensitivity analyses show stable pooled estimates | Low heterogeneity  (I² = 0.04%) | High heterogeneity  (I² = 76.27%) |
| **Indirectness** | Many retrospective studies (potential selection bias)  AI tested in retrospect and not in real-time  Differences between study population vs. real-world patients, and between study methods and clinical protocols (eg only 4CV assessed vs five axial views as recommended by guidelines) | Many retrospective studies (potential selection bias)  AI tested in retrospect and not in real-time  Differences between study population vs. real-world patients, and between study methods and clinical protocols (eg only 4CV assessed vs five axial views as recommended by guidelines) | Many retrospective studies (potential selection bias)  AI tested in retrospect and not in real-time  Differences between study population vs. real-world patients and study methods and clinical protocols (eg only 4CV assessed vs five axial views as recommended by guidelines) |
| **Imprecision** | Narrow confidence intervals for sensitivity and specificity | Narrow confidence intervals for sensitivity and specificity | Narrow confidence intervals for sensitivity and specificity |
| **Publication Bias** | Deek’s test not significant, this no strong evidence of publication bias | Not assessed as the number of studies included in the subgroup are < 10 | Not assessed as the number of studies included in the subgroup are < 10 |
| **Final GRADE Certainty** | **LOW**  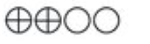 | **LOW**  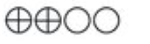 | **LOW**  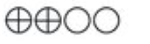 |

**Supplementary Figure 1 – Training with normal fetal heart data**

**
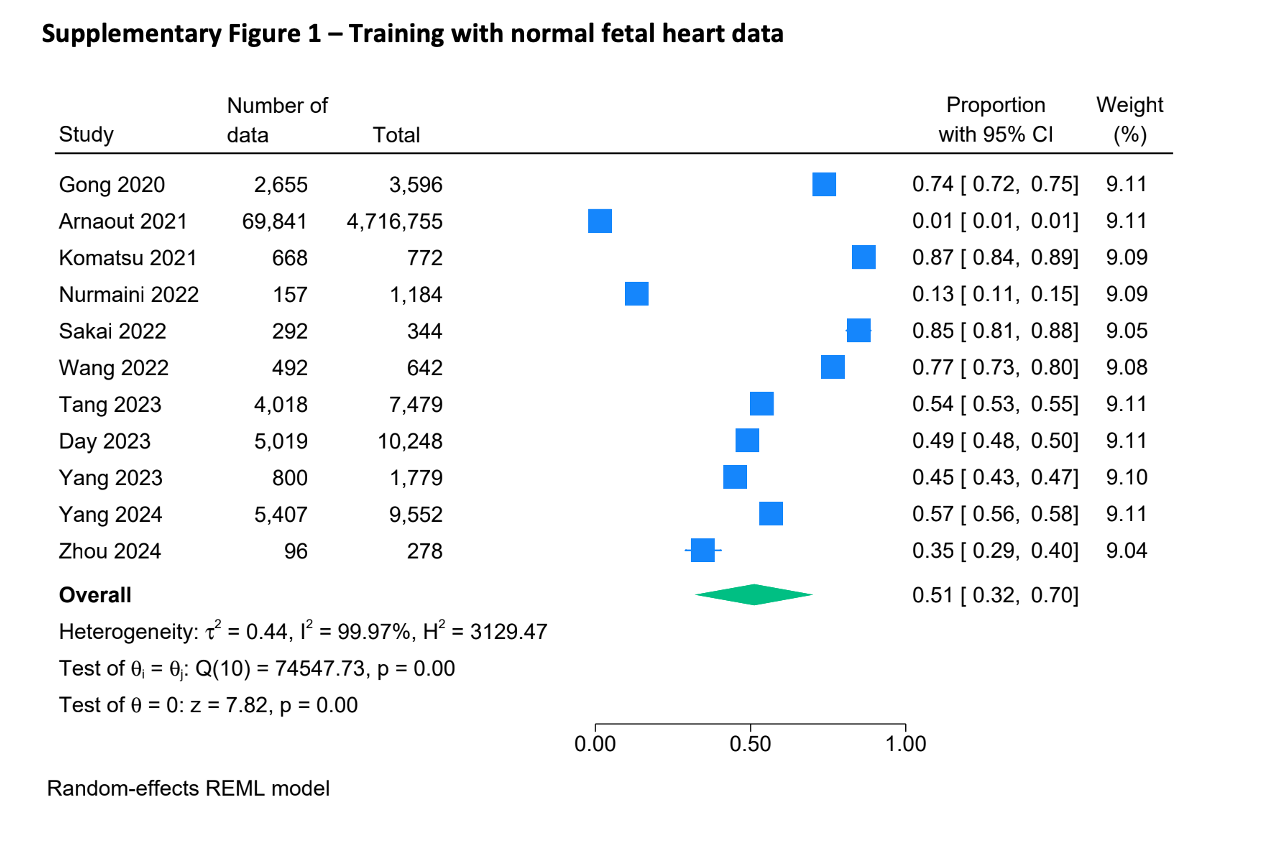
**

**Supplementary Figure 2 – Training with abnormal fetal heart data**


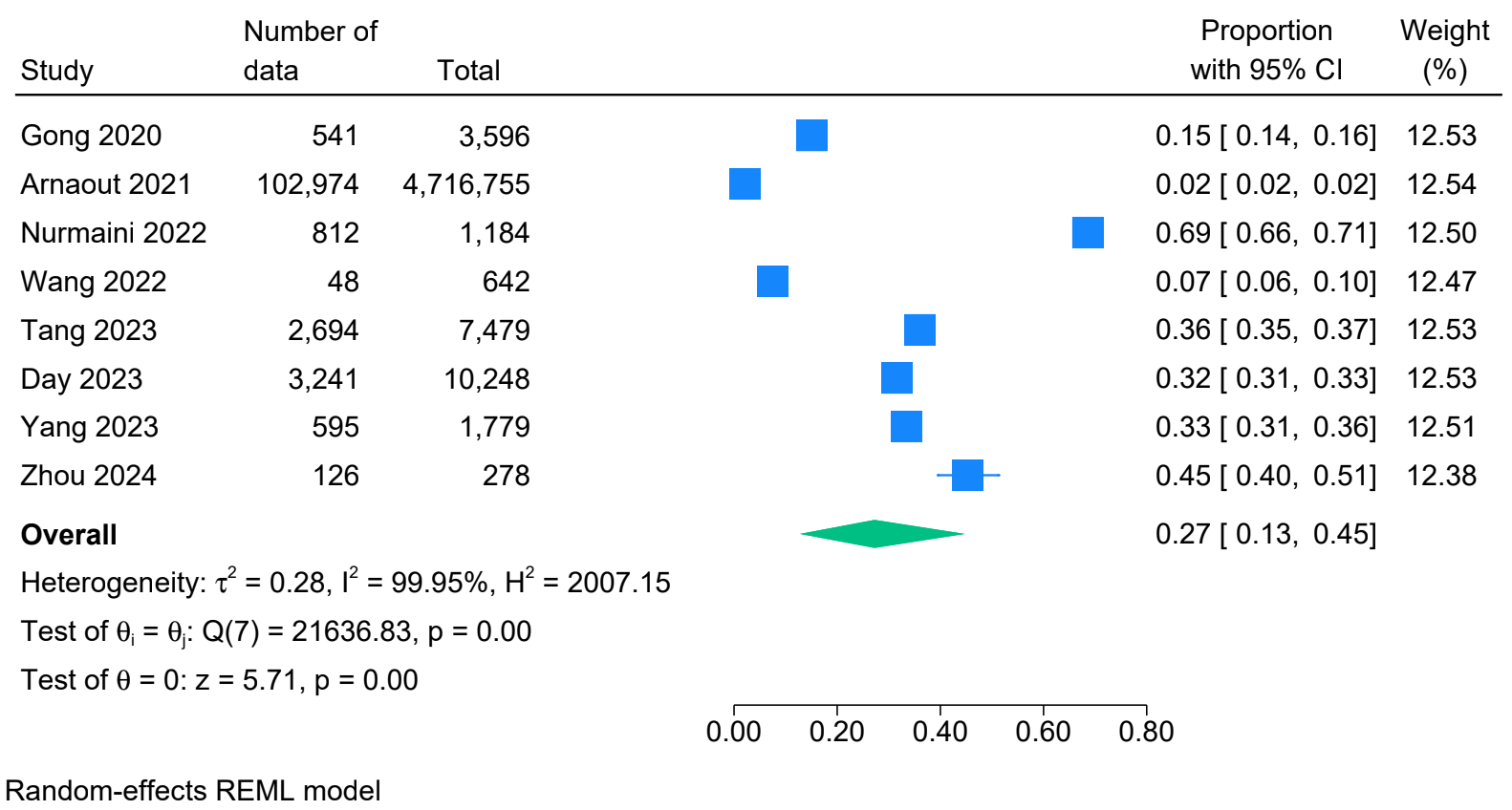


**Supplementary Figure 3 – Validation with normal fetal heart data**


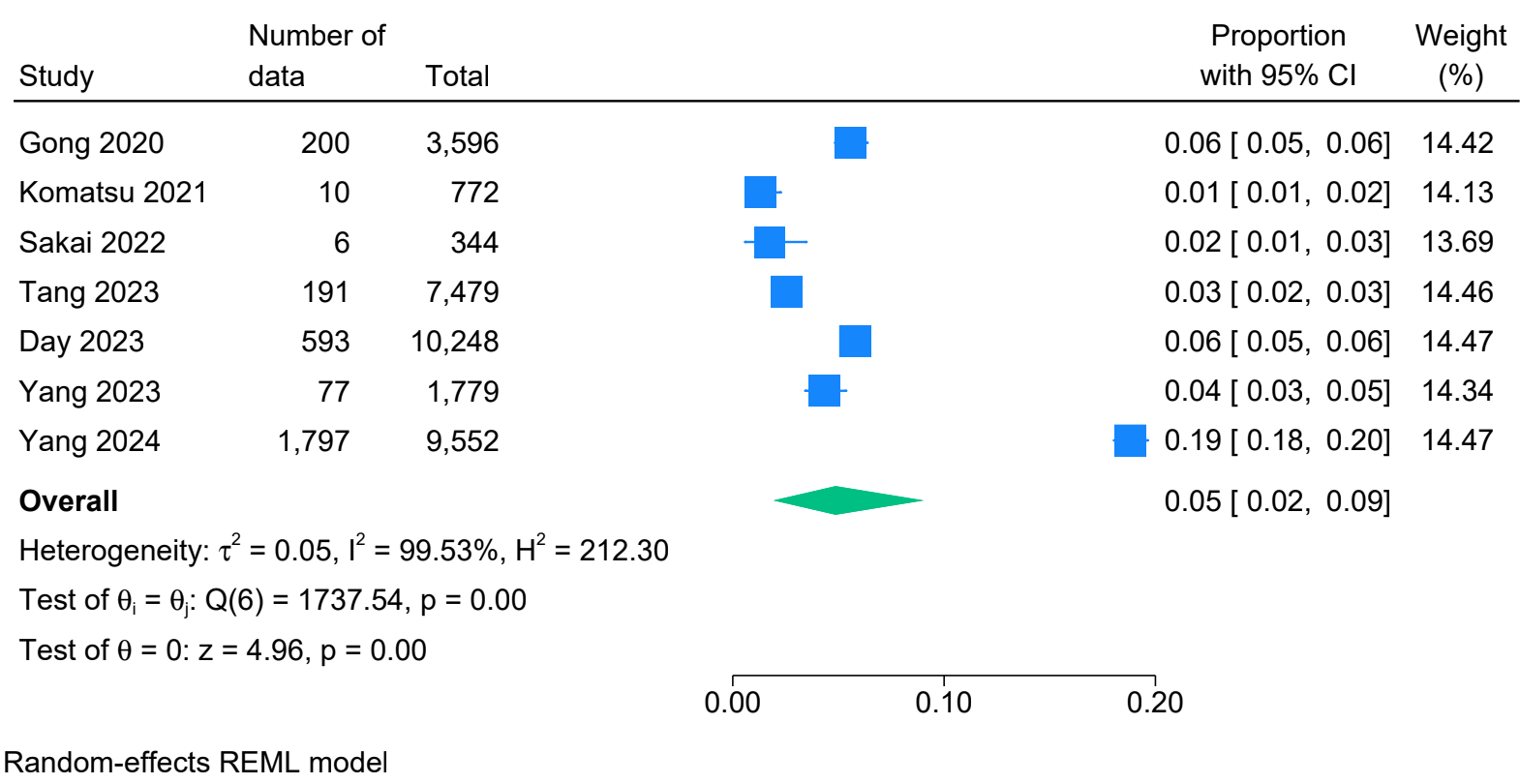


**Supplementary Figure 4 – Validation with abnormal fetal heart data**

**
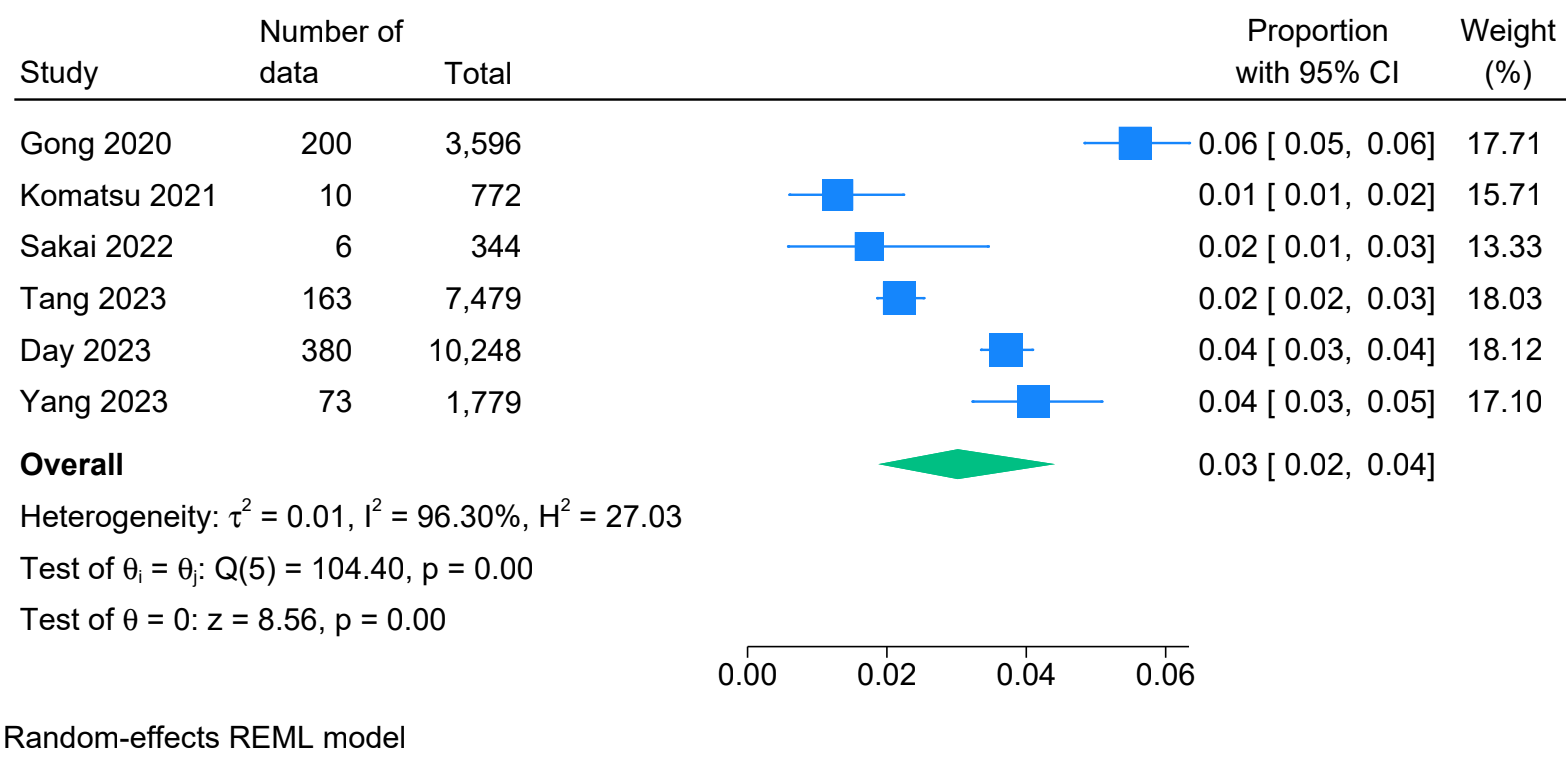
**

**Supplementary Figure 5 – Testing with normal fetal heart data**

**
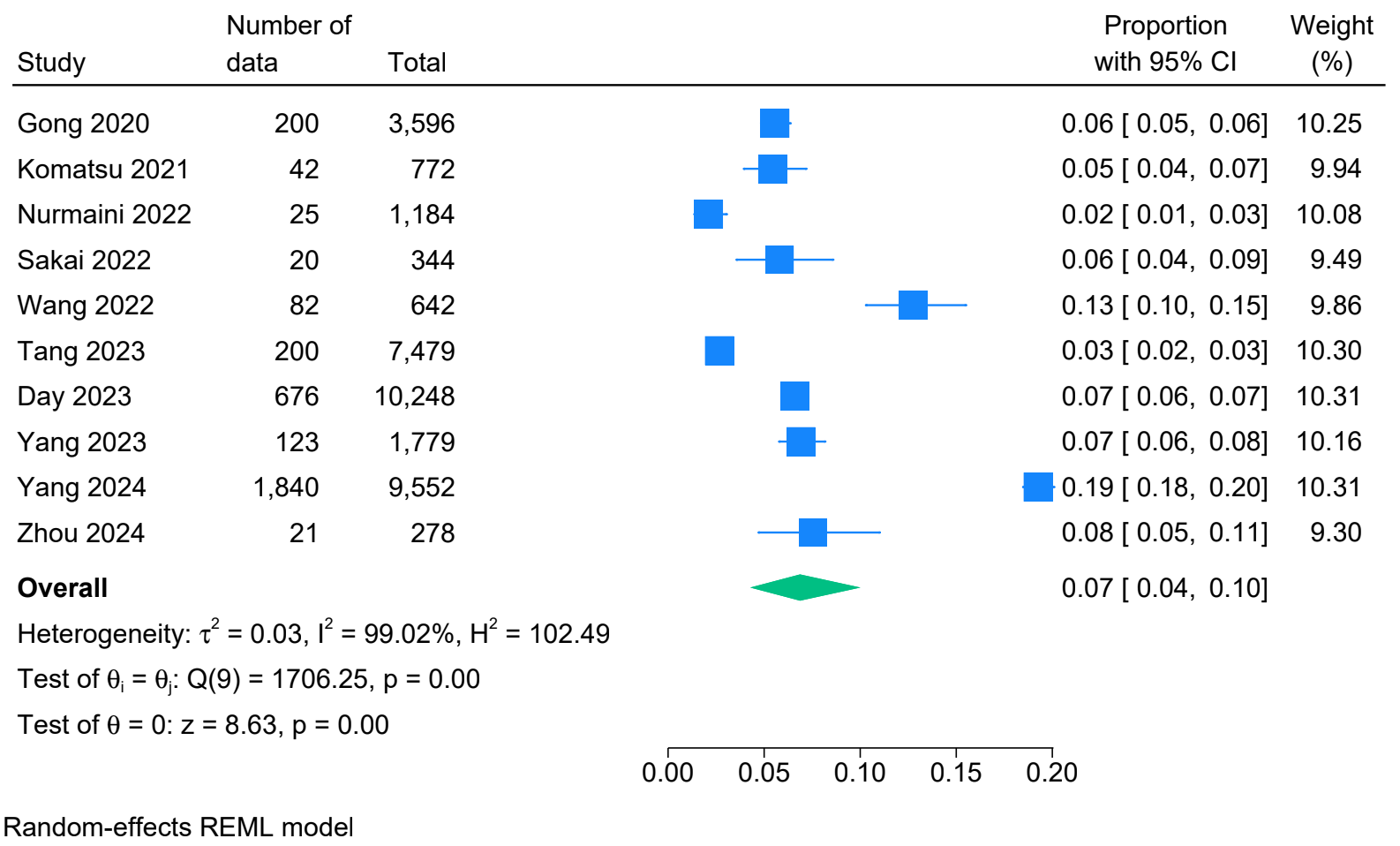
**

**Supplementary Figure 6 – Testing with abnormal fetal heart data**

**
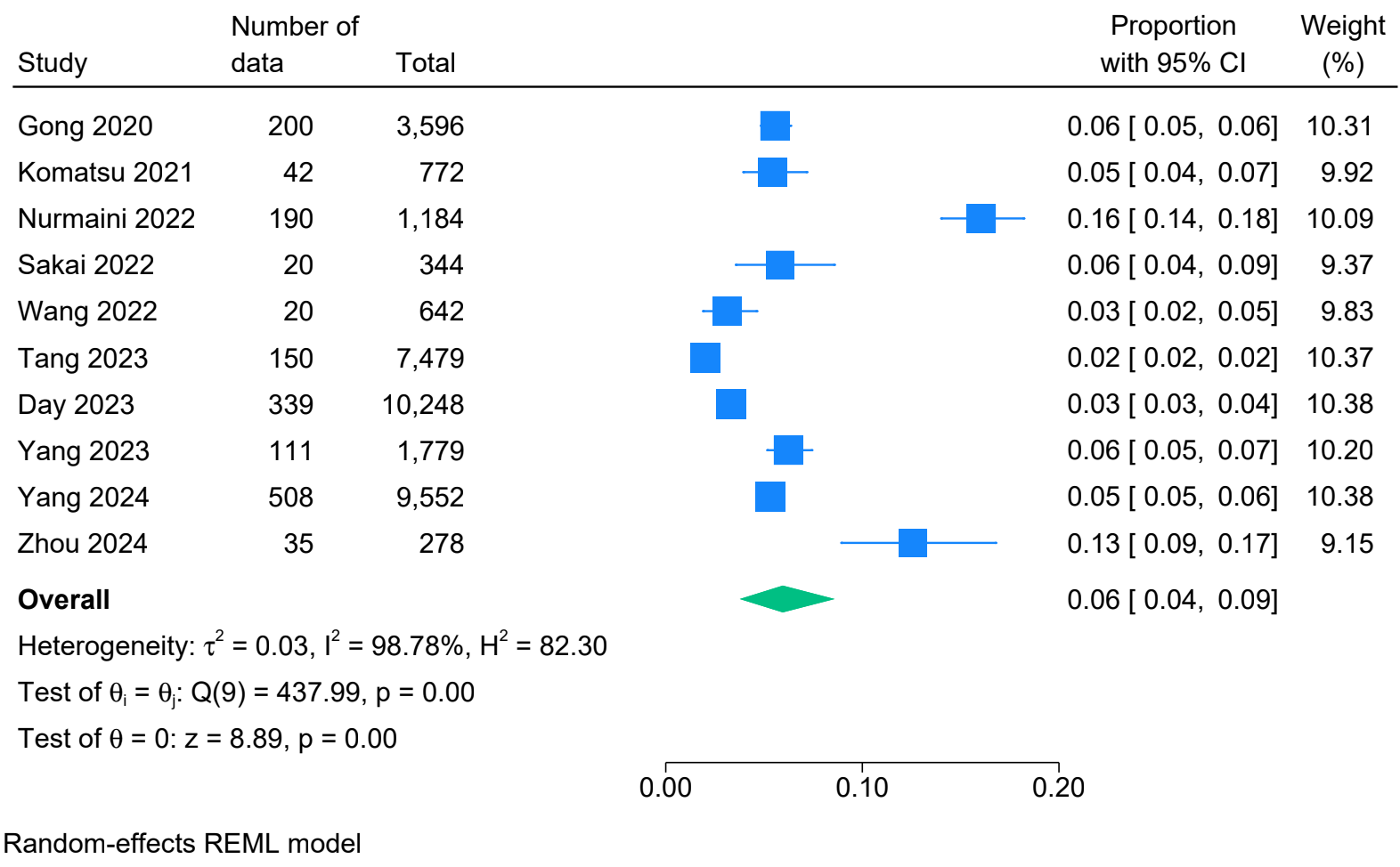
**


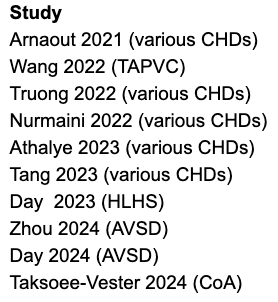
**Supplementary Figure 7a – Forest plot and overall pooled specificity and sensitivity for AI classification of normal vs abnormal hearts**

**
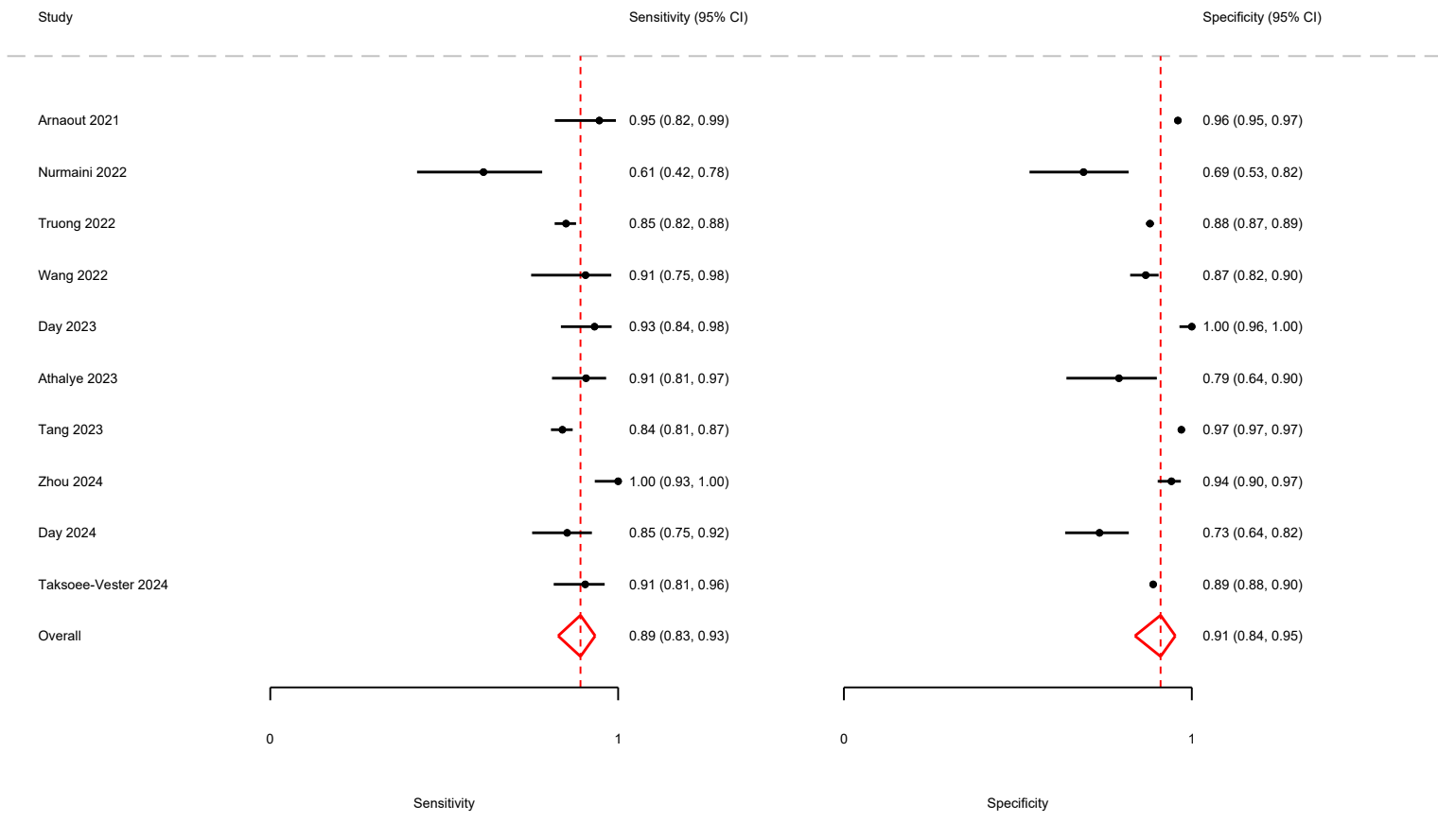
**

**Supplementary Figure 7b – sROC for overall AI classification of normal vs abnormal hearts**

**
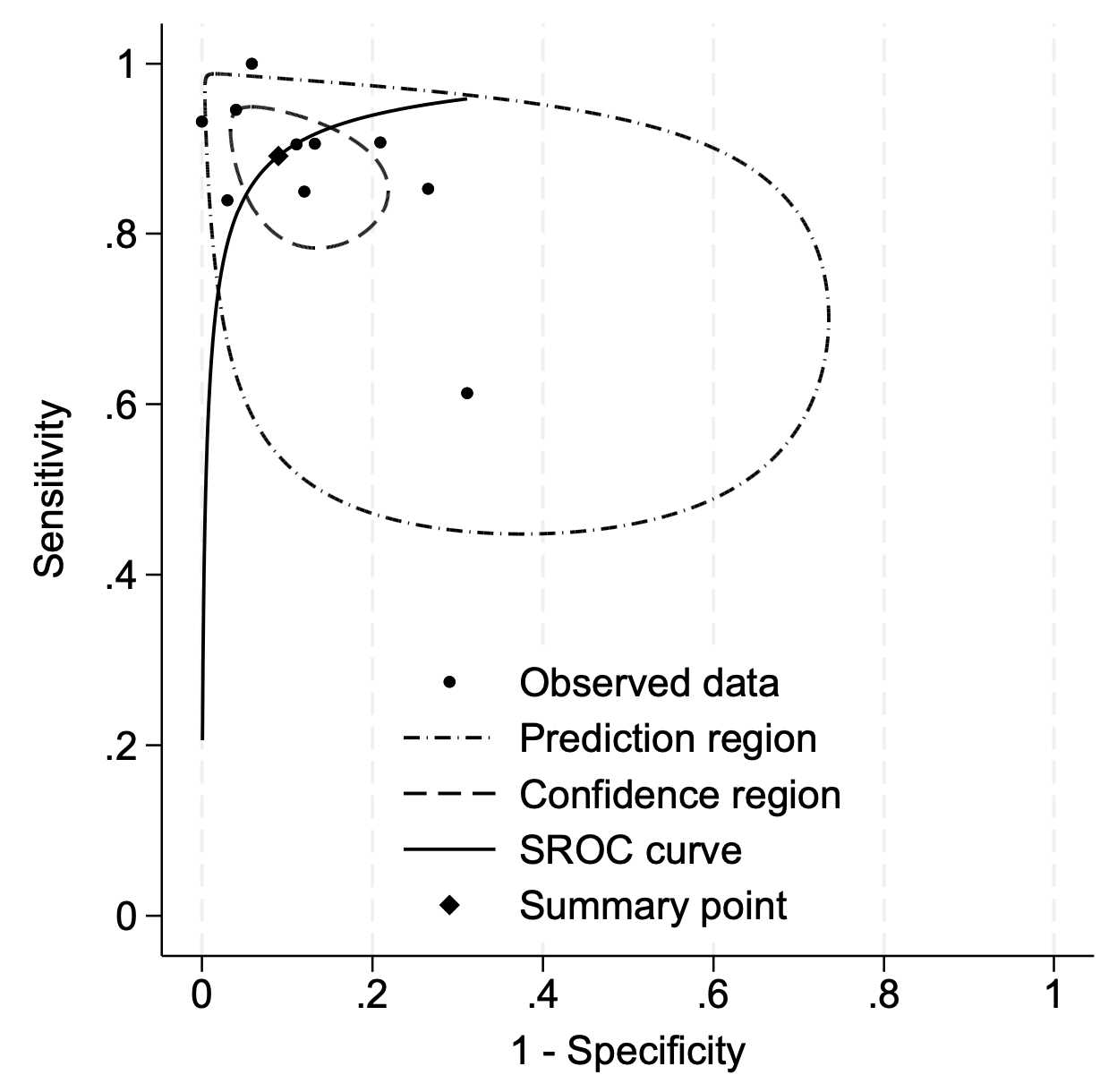
**

**Supplementary Figure 8a – Forest plot and pooled specificity and sensitivity for AI classification of normal vs abnormal hearts (model tested with various CHDs)**

**
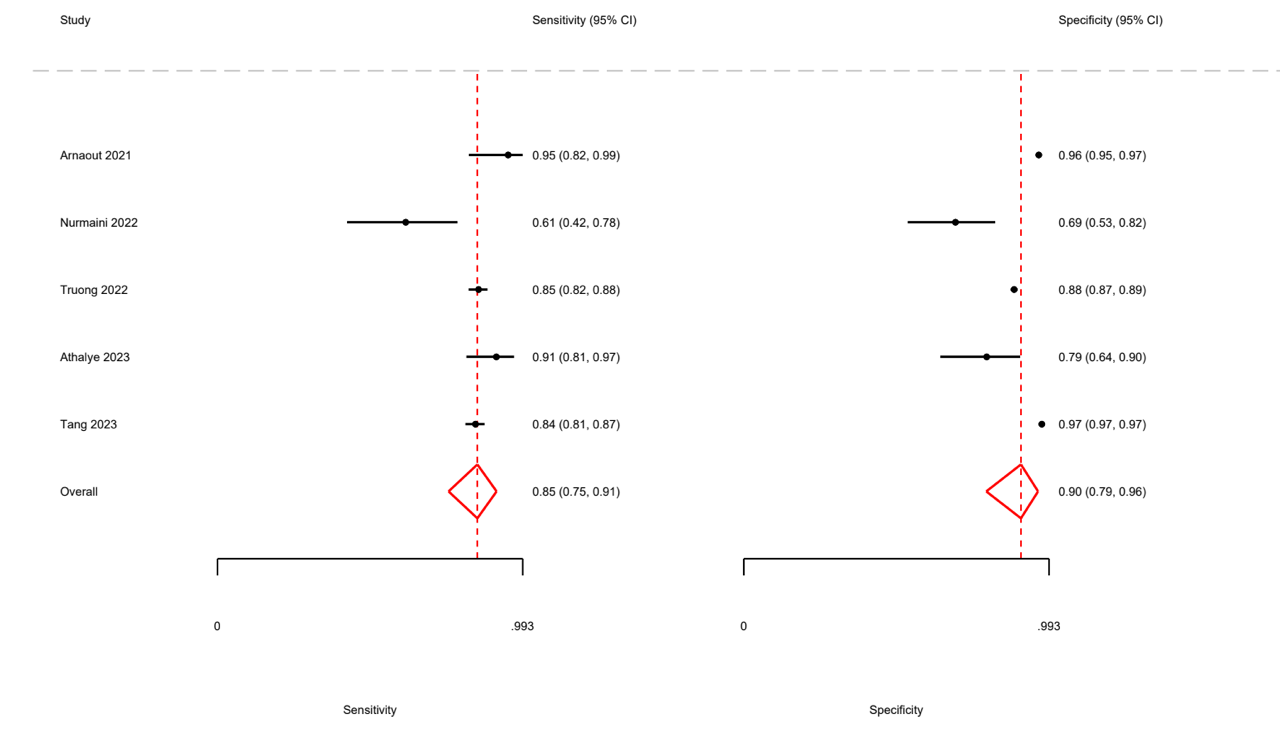
**

**
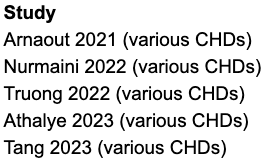
**

**Supplementary Figure 8b – sROC for AI classification of normal vs abnormal hearts (model tested with various CHDs )**


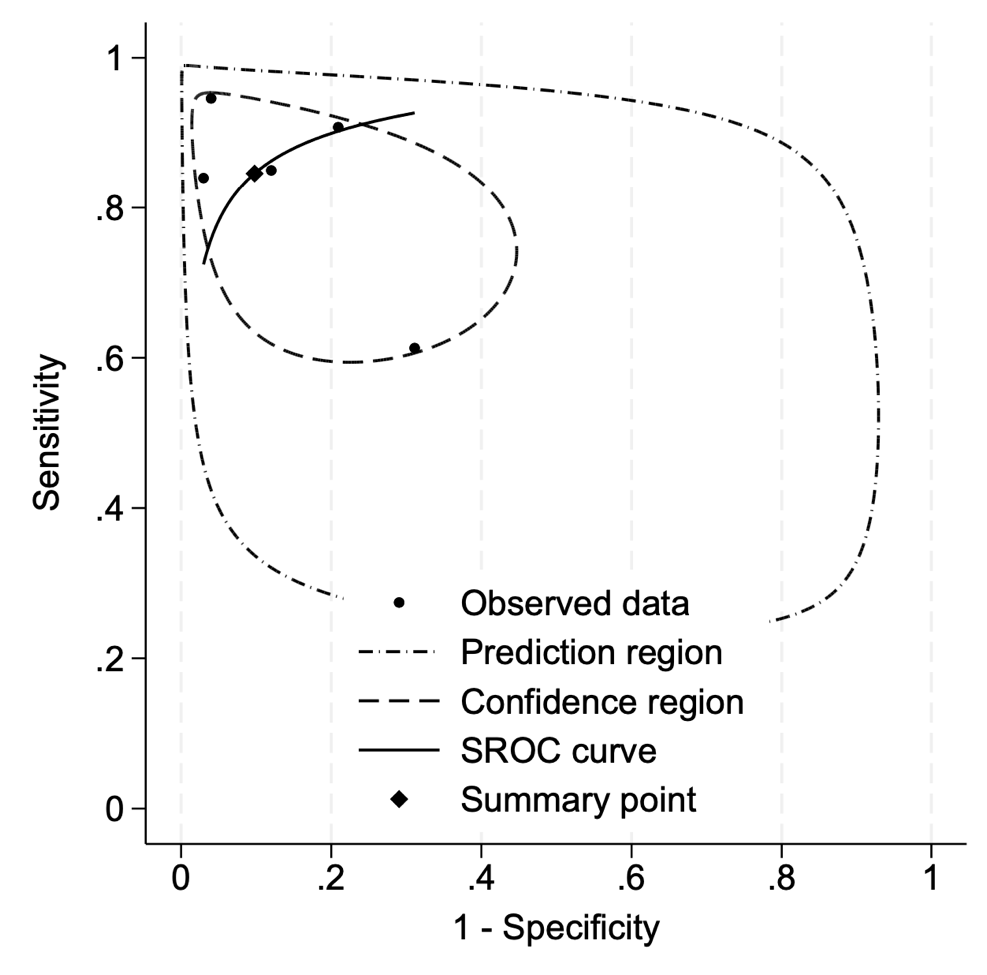


**Supplementary Figure 9a – Forest plot and pooled specificity and sensitivity for AI classification of normal vs abnormal hearts (model tested with a specific cardiac abnormality)**


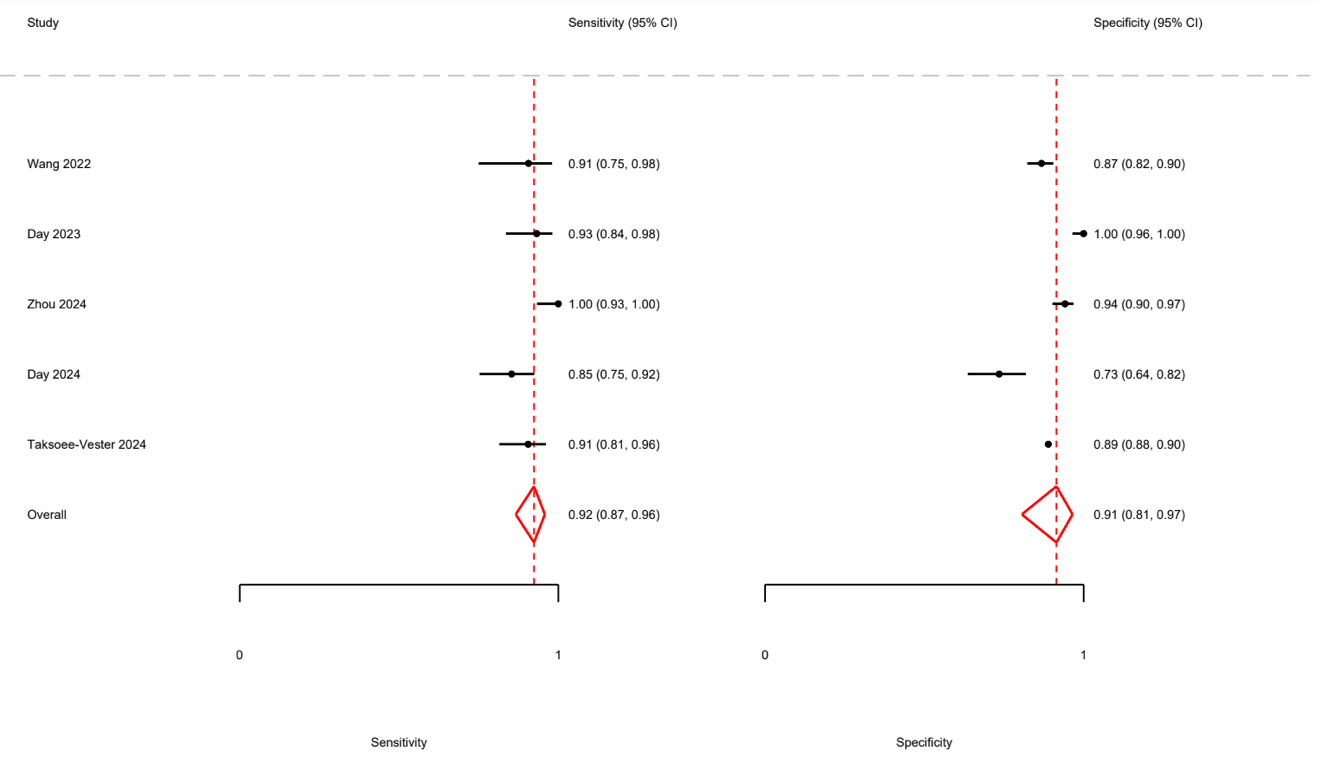


**
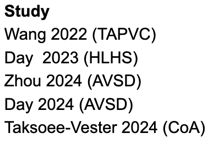
**

**Supplementary Figure 9b – sROC for AI classification of normal vs abnormal hearts (model tested with a specific cardiac abnormality)**


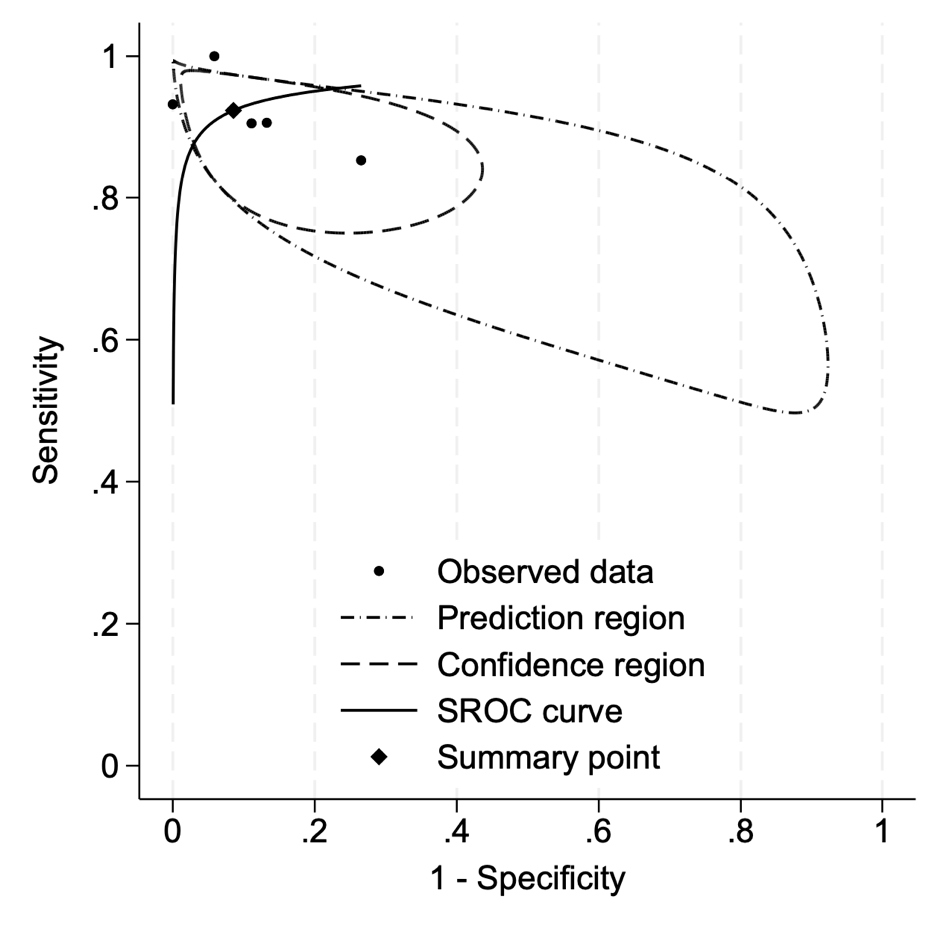

Supplement: Supplementary Tables and Figures — Supplementary Figure 1. Training with normal fetal heart data. Forest plot showing the proportion of normal fetal heart data used for training across included studies. Supplementary Figure 2. Training with abnormal fetal heart data. Forest plot showing the proportion of abnormal fetal heart data used for training across included studies. Supplementary Figure 3. Validation with normal fetal heart data. Forest plot showing the proportion of normal fetal heart data used for validation across included studies. Supplementary Figure 4. Validation with abnormal fetal heart data. Forest plot showing the proportion of abnormal fetal heart data used for validation across included studies. Supplementary Figure 5. Testing with normal fetal heart data. Forest plot showing the proportion of normal fetal heart data used for testing across included studies. Supplementary Figure 6. Testing with abnormal fetal heart data. Forest plot showing the proportion of abnormal fetal heart data used for testing across included studies. Supplementary Figure 7. a Forest plot and overall pooled specificity and sensitivity for AI classification of normal vs abnormal hearts. CHDs, congenital heart defects; TAPVC, total anomalous pulmonary venous connection; HLHS, hypoplastic left heart syndrome; AVSD, atrioventricular septal defect; CoA, coarctation of the aorta. b sROC for overall AI classification of normal vs abnormal hearts Supplementary Figure 8. a Forest plot and pooled specificity and sensitivity for AI classification of normal vs abnormal hearts (model tested with various CHDs). b sROC for AI classification of normal vs abnormal hearts (model tested with various CHDs).Supplementary Figure 9. a Forest plot and pooled specificity and sensitivity for AI classification of normal vs abnormal hearts (model tested with a specific cardiac abnormality) TAPVC, total anomalous pulmonary venous connection; HLHS, hypoplastic left heart syndrome; AVSD, atrioventricular septal defect; CoA, coarctation of [file mmc1.docx]
