# Supplementary material for: Artificial intelligence-enabled prenatal ultrasound for the detection of fetal cardiac abnormalities: a systematic review and meta-analysis
Source: eClinicalMedicine. 2025 May 30;84:103250. doi: 10.1016/j.eclinm.2025.103250 (PMC12273734; doi:10.1016/j.eclinm.2025.103250)
Supplement: Appendix [file mmc2.pdf]

Search Strategy	CINAHL	
S1	TX (((artificial* intelligen* or AI or machine learn* or deep learn* or transfer learn* or neural network* or intelligent navigation or data mining or segmentation)))	76,626
S2	TX ((fetal or foetal or foetus* or fetus* or prenatal or antenatal or in utero or uterine or unborn or second trimester or third trimester or 2nd trimester or 3rd trimester or (16 weeks N3 pregnan*)))	193,823
S3	TX ((echocardiograph* or ultrasound* or scan* or imag*))	800,372
S4	TX ((((heart* or cardiac) N3 (Defect* or anomal* or abnormal* or tumor* or tumour* or block or malformation)) or cardiology or congenital or (coarctation N3 aorta) or (transposition N3 great N3 (arter* or vessel*)) or anomalous pulmonary venous connection* or aortic arch interruption* or aortic stenosis* or arrhythmia* or asplenia syndrome* or atrial isomerism* or atrioventricular septal defect* or cardiomyopath* or common arterial trunk* or congenitally corrected transposition of the great arteries* or cor triatriatum* or coronary vessel malformation* or criss cross atrioventricular relationship* or cyanotic heart disease* or dextrocardia* or double aortic arch* or double inlet left ventricle* or double outlet right ventricle* or dysplastic tricuspid valve* or Ebstein anomal* or Ebstein anomaly* or ectopia cordis* or foramen ovale* or heart atrium septum defect* or heart left right shunt* or heart right left shunt* or heart right ventricle double outlet* or heart septum defect* or heart single ventricle* or heart ventricle septum defect* or heterotax* or hypoplastic left heart syndrome* or intact interventricular septum* or isolated left isomerism* or left ventricular outflow tract obstruction* or levocardia* or pulmonary atresia* or pulmonary stenosis* or right aortic arch* or situs inversus* or (tetralogy N2 Fallot) or (tricuspid N3 atresia) or truncus arteriosus* or univentricular* or ventricular noncompaction* or ventricular septal defect* or ventriculoatrial shunt*).)	347,737
S5	S1 AND S2 AND S3 AND S4	107

**Search Strategy**	https://www.cochranelibrary.com/advanced-search/search-manager?search=7486085	
ID	Search	Hits
#1	[mh "Artificial Intelligence"] OR [mh ^"Image Processing, Computer-Assisted"] in Trials	5455
#2	((artificial* NEAR/2 intelligen*) OR AI OR (machine NEAR/2 learn*) OR (deep NEAR/2 learn*) OR (transfer NEAR/2 learn*) OR (neural NEAR/2 network*) OR (intelligent NEAR/2 navigation) OR (data NEAR/2 mining) OR segmentation):ti,ab,kw	13251
#3	#1 or #2	16805
#4	[mh "Fetal Diseases"] OR ([mh ^"Pregnancy Trimester, Second"] OR [mh ^"Pregnancy Trimester, Third"]) OR [mh Fetus]	5085

#5	(fetal or foetal or foetus* or fetus* or prenatal or antenatal or in utero or uterine or unborn or second trimester or third trimester or 2nd trimester or 3rd trimester or (16 weeks NEAR/3 pregnan*)):ti,ab,kw	50144
#6	#4 or #5	50894
#7	[mh "Diagnostic Imaging"]	69471
#8	(echocardiograph* or ultrasound* or scan* or imag*):ti,ab,kw	191960
#9	#7 or #8	204852
#10	#3 and #6 and #9 in Trials	138

	https://ovidsp.ovid.com/ovidweb.cgi?T=JS&NEWS=N&PAGE=main&SHAREDSEARCHID=2RelE4CTdhKhJAaS297UP7vXPpSxAh0NEA4HaU86LXZ8e1dkPoy2mpYwU8j5US4Jq	
Embase 1974 to present		
1	exp artificial intelligence/ or exp machine learning/ or exp image segmentation/ or exp imaging algorithm/	548503
2	(artificial* intelligen* or AI or machine learn* or deep learn* or transfer learn* or neural network* or intelligent navigation or data mining or segmentation).ti,ab,kf.	438200
3	1 or 2	711771
4		278733
5	(fetal or foetal or foetus* or fetus* or prenatal or antenatal or in utero or unborn or first trimester or 1st trimester or ((16 weeks or Early) adj3 pregnan*) or congenital*).ti,ab,kf.	997008
6	4 or 5	1048022
7	exp echocardiography/ or exp diagnostic imaging/	724857
8	(echocardiograph* or ultrasound* or scan* or imag*).ti,ab,kf.	3498424
9	7 or 8	3767587
10	6 and 9	166997
11	fetus echography/	31413

12	10 or 11	17926<br>3
13	exp congenital heart disease/	20432<br>3
14	(((heart* or cardiac) adj3 (Defect* or anomal* or abnormal* or tumor* or tumour* or block or malformation)) or (coarctation adj3 aorta) or (transposition adj3 great adj3 (arter* or vessel*)) or anomalous pulmonary venous connection* or aortic arch interruption* or aortic stenosis* or arrhythmia* or arrhythmogenic right ventricular dysplasia* or asplenia syndrome* or atrial isomerism* or atrioventricular septal defect* or Barth syndrome* or cardiomyopath* or common arterial trunk* or congenitally corrected transposition of the great arteries* or cor triatriatum* or coronary vessel malformation* or criss cross atrioventricular relationship* or cyanotic heart disease* or desminopathy* or dextrocardia* or double aortic arch* or double inlet left ventricle* or double outlet right ventricle* or ductus arteriosus* or ductus arteriosus obliteration* or dysplastic tricuspid valve* or Ebstein anomal* or Ebstein anomaly* or ectopia cordis* or Eisenmenger complex* or endocardial cushion defect* or foramen ovale* or heart atrium septum defect* or heart left right shunt* or heart right left shunt* or heart right ventricle double outlet* or heart septum defect* or heart single ventricle* or heart ventricle septum defect* or heterotax* or Holt Oram syndrome* or hypoplastic left heart syndrome* or intact interventricular septum* or isolated left isomerism* or Kartagener syndrome* or left ventricular outflow tract obstruction* or LEOPARD syndrome* or levocardia* or McKusick Kaufman syndrome* or myocardial bridging* or Naxos disease* or pulmonary atresia* or pulmonary stenosis* or right aortic arch* or situs inversus* or (tetralogy adj2 Fallot) or (tricuspid adj3 atresia) or truncus arteriosus* or univentricular* or velocardiofacial syndrome* or ventricular noncompaction* or ventricular septal defect* or ventricular tunnel* or ventriculoatrial shunt*).ti,ab,kf.	49440<br>3
15	13 or 14	58696<br>3
**16**	**3 and 12 and 15**	**733**

**Search Strategy**		
	https://ovidsp.ovid.com/ovidweb.cgi?T=JS&NEWS=N&PAGE=main&SHAREDSEARCHID=4w80G5C83dA60XZ2prJPfqivce7Eju4eLZ2lo04uqTinbVnAc0nikSTCifDoT6Z2D	
	**Medline (Ovid MEDLINE® Epub Ahead of Print, In-Process & Other Non-Indexed Citations, Ovid MEDLINE® Daily and Ovid MEDLINE®) 1946 to present**	
1	exp Artificial Intelligence/ or Image Processing, Computer-Assisted/	332<br>786

2	(artificial* intelligen* or AI or machine learn* or deep learn* or transfer learn* or neural network* or intelligent navigation or data mining or segmentation).ti,ab,kf.	361659
3	1 or 2	562444
4	exp Fetal Diseases/ or (Pregnancy Trimester, Second/ or Pregnancy Trimester, Third/) or exp Fetus/	248895
5	(fetal or foetal or foetus* or fetus* or prenatal or antenatal or in utero or uterine or unborn or second trimester or third trimester or 2nd trimester or 3rd trimester or (16 weeks adj3 pregnan*)).ti,ab,kf.	626739
6	4 or 5	724806
7	exp Diagnostic Imaging/	2978988
8	(echocardiograph* or ultrasound* or scan* or imag*).ti,ab,kf.	2514774
9	7 or 8	4292882
10	6 and 9	117440
11	exp Prenatal Diagnosis/	82911
12	10 or 11	157603
13	exp Heart Defects, Congenital/	173319

14	(((heart* or cardiac) adj3 (Defect* or anomal* or abnormal* or tumor* or tumour* or block or malformation)) or cardiology or congenital or (coarctation adj3 aorta) or (transposition adj3 great adj3 (arter* or vessel*)) or anomalous pulmonary venous connection* or aortic arch interruption* or aortic stenosis* or arrhythmia* or asplenia syndrome* or atrial isomerism* or atrioventricular septal defect* or cardiomyopath* or common arterial trunk* or congenitally corrected transposition of the great arteries* or cor triatriatum* or coronary vessel malformation* or criss cross atrioventricular relationship* or cyanotic heart disease* or dextrocardia* or double aortic arch* or double inlet left ventricle* or double outlet right ventricle* or dysplastic tricuspid valve* or Ebstein anomal* or Ebstein anomaly* or ectopia cordis* or foramen ovale* or heart atrium septum defect* or heart left right shunt* or heart right left shunt* or heart right ventricle double outlet* or heart septum defect* or heart single ventricle* or heart ventricle septum defect* or heterotax* or hypoplastic left heart syndrome* or intact interventricular septum* or isolated left isomerism* or left ventricular outflow tract obstruction* or levocardia* or pulmonary atresia* or pulmonary stenosis* or right aortic arch* or situs inversus* or (tetralogy adj2 Fallot) or (tricuspid adj3 atresia) or truncus arteriosus* or univentricular* or ventricular noncompaction* or ventricular septal defect* or ventriculoatrial shunt*).ti,ab,kf.	624885
15	13 or 14	701388
**16**	**3 and 12 and 15**	**346**
